# Functionalization of Cotton by Thermoresponsive Polymer Brushes for Potential Use as Smart Dressings

**DOI:** 10.1021/acsapm.5c00534

**Published:** 2025-04-18

**Authors:** Izabela Zaborniak, Michał Sroka, Kamil Wilk, Anna Cieślik, Joanna Raczkowska, Kaja Spilarewicz, Natalia Janiszewska, Kamil Awsiuk, Karol Wolski, Kinga Pielichowska, Paweł Błoniarz, Katarzyna Kisiel, Magdalena Bednarenko, Krzysztof Matyjaszewski, Paweł Chmielarz

**Affiliations:** † Department of Physical Chemistry, Faculty of Chemistry, Rzeszow University of Technology, al. Powstańców Warszawy 6, Rzeszów 35-959, Poland; ‡ Department of Chemistry, Carnegie Mellon University, 4400 Fifth Ave., Pittsburgh, Pennsylvania 15213, United States; § Doctoral School of Exact and Natural Sciences, Jagiellonian University, Łojasiewicza 11, Kraków 30-348, Poland; ∥ Faculty of Physics, Astronomy and Applied Computer Science, M. Smoluchowski Institute of Physics, Jagiellonian University, Łojasiewicza 11, Kraków 30-348, Poland; ⊥ Faculty of Chemistry, Jagiellonian University, Gronostajowa 2, Kraków 30-387, Poland; # Department of Glass Technology and Amorphous Coatings, Faculty of Materials Science and Ceramics, AGH University of Krakow, Al. Mickiewicza 30, Kraków 30-059, Poland; ∇ Doctoral School of the Rzeszow University of Technology, Rzeszow University of Technology, al. Powstańców Warszawy 8, Rzeszów 35-959, Poland

**Keywords:** cotton, polymer brushes, SARA ATRP, thermoresponsive surfaces, smart dressing

## Abstract

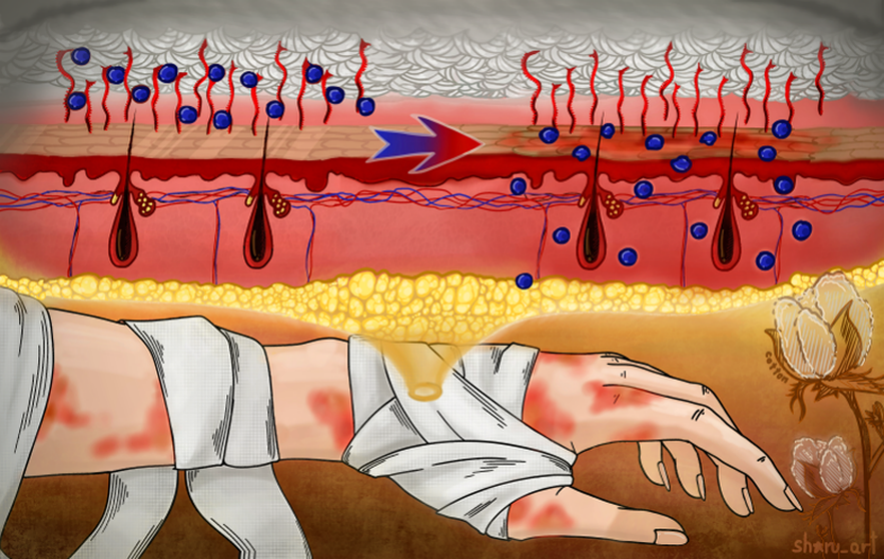

Cotton is the most widely used dressing material due to its universal availability, affordability, high biodegradability, and ease of recyclability. Modern and advanced techniques for controlled polymer grafting onto its surface enhance and precisely tailor cotton’s properties. These improvements contribute to the healing process by preventing adhesion to wounds, facilitating the absorption of body fluids, and enabling the design of innovative dressings capable of the controlled release of active substances. Therefore, this study presents the grafting of thermoresponsive polymer brushes composed of di(ethylene glycol) methyl ether methacrylate (DEGMA) and poly(ethylene glycol) methyl ether methacrylate (OEGMA, *M_n_* ∼ 500) onto a cotton surface using surface-initiated supplemental activator and reducing agent atom transfer radical polymerization (SI-SARA ATRP). By precisely adjusting the composition of DEGMA and OEGMA_500_, we achieved precise control over the polymer layer’s lower critical solution temperature (LCST) behavior. The LCST of the copolymers formed in the reaction mixture in the presence of the functionalized surface was analyzed via transmittance measurements. Furthermore, the thermoresponsive properties of the polymer layer grafted onto the cotton surface were evaluated through water contact angle (WCA) measurements at varying temperatures. In addition, the temperature-dependent protein adsorption of the polymer-functionalized cotton was examined to assess the potential dressing’s adherence to wounds. Finally, the resulting materials were tested for residual copper content and cytotoxicity.

## 1 Introduction

Among the various materials available in the textile industry, cotton is the most extensively used fiber, accounting for more than 80% of global natural fiber production. It is the second most produced fiber overall, following polyester.^1^ Cotton’s widespread cultivation and the ease with which it can be processed contribute to its use in more than 100 countries worldwide.^2^ Additionally, cotton is recognized as one of the purest forms of cellulose, making it highly biodegradable and easily recyclable, which supports its application in a broad range of textile industries. The softness of cotton makes it a preferred choice for clothing, providing a comfortable experience for consumers and solidifying its popularity in the fashion industry. Once processed, cotton becomes a versatile material capable of absorbing fluids, such as blood and wound exudates. Its large surface area, porosity, and gas permeability render cotton wound dressings and bandages particularly suitable for medical applications.^3^ Although gauze is the most commonly used wound dressing in clinical practice, its application faces several challenges. One major issue is the absorption of the wound exudate into the gauze. While this absorption helps manage moisture, it also provides an environment rich in nutrients and moisture, which can promote bacterial growth and lead to serious wound infections. Another significant drawback is the tendency of gauze to adhere to the wound after absorbing exudate. This adherence can cause secondary injury during dressing changes, making the process painful and potentially damaging to the healing tissue. Additionally, this property makes gauze unsuitable for burn wounds, where minimal adherence is essential to prevent further tissue damage.^4,5^

Considering the disadvantages of cotton gauze dressings, they may have a more beneficial application in the treatment of closed skin edema, including after burns or bites, which are accompanied by redness, local skin irritation, and swelling.^6^ As the severity of an injury increases, more advanced treatment methods become necessary, going beyond the basic use of moisture gauzes, bandages, or hydrogels.^4,5^ Whether using wound dressing, there is also the opportunity to incorporate drugs and antibiotics essential for proper wound healing.^7^ This is particularly crucial for wounds caused by insect bites. While most insect bites result in harmless, temporary changes to the skin, some can lead to serious skin infections that require appropriate antibiotic treatment. Traditional methods for delivering predetermined doses of medication include the application of creams, gels, or drug-infused dressings.^8^ However, these approaches often necessitate frequent dressing changes, which can cause discomfort and pain for the patient.

Therefore, the primary challenge for modern dressings is to create low-adherence materials that can effectively manage the controlled release of antibiotics or drugs in response to environmental changes. These changes typically include variations in pH, such as the elevated pH of body fluids in open wounds, or fluctuations in temperature, particularly in burn or insect bite wounds.^9^

One effective way to precisely tailor the properties of surfaces, both flat and porous, organic and inorganic, is to functionalize them with covalently attached polymers in a controlled manner.^10−14^ The process can utilize reversible deactivation radical polymerization (RDRP), specifically surface-initiated atom transfer radical polymerization (SI-ATRP), to graft polymer chains with well-defined structures and tailored properties.^10,13,15−17^ One methodology is the “*grafting from*” approach, which involves covalently attaching an initiator to the surface via esterification, enabling in situ polymerization in a controlled manner directly from the surface. The reversible reaction between the alkyl-halide macroinitiator and the transition-metal-based catalytic complex ensures an equilibrium between dormant alkyl halides and active radicals, enabling the formation of polymer brushes on the substrate surface.^10,18−21^ This method facilitates achieving a high grafting density of polymer brushes on the surface, as steric hindrance appears to be low. Initiators may either be originally present on the surface or introduced during additional functionalization reactions to form α-bromoesters or α-bromoamides.^11,22−24^

Up to now, surface-initiated activator regenerated by electron transfer (SI-ARGET) ATRP was used for grafting poly(2-(dimethylamino)ethyl methacrylate) (PDMAEMA) from cotton surfaces to create low-adherent wound dressings.^25^ In another study, cotton was functionalized by poly(*N*-isopropylacrylamide) (PNIPAM) using SI-ATRP to develop a temperature-responsive moisture management system regulated by body temperature.^26^ While the other work presents surface-initiated ATRP of OEGMA_500_ from cotton in the presence of a Zn^0^ plate according to the supplemental activator and reducing agent (SARA) ATRP, however thermoresponsive properties of the prepared surface were not further studied.^27^

Therefore, we decided to explore the precise tailoring of the thermoresponsive properties of the polymer layer onto the cotton surface, presenting a novel type of cotton-based wound dressing incorporating a potential temperature-activated controlled drug release mechanism activated by temperature changes. The unique feature of the polymers used in this research is their ability to undergo phase transitions at LCST, slightly above a normal human body temperature. The concept of grafting thermosensitive polymer brushes from the surface to encapsulate and release active substances has already been described in the literature. For instance, one of the most recent studies presents the fabrication and validation of functional titanium-based implants with a triggered antibiotic release function, achieved through an intelligent polymer coating based on thermoresponsive poly(di(ethylene glycol) methyl ether methacrylate) (PDEGMA).^28^ This approach enabled the controlled and thermally triggered release of the antibiotic levofloxacin at the wound site. The antibiotic-loaded polymer brushes were analyzed in terms of their thickness, antibiotic loading capacity, and temperature-dependent release behavior. Moreover, there have been few studies on PNIPAM-based polymer brushes grafted from surfaces e.g., from polyethylene surfaces by functionalizing implant devices to sustained release of R-hirudin as a model drug to prevent restenosis from the functionalized surface.^29^ In addition, thermoresponsive implantable PNIPAM-based hydrogels were applied to endotracheal tubes by Jones et al.^30^ The faster release of metronidazole at a temperature above the LCST was employed to reduce mortality related to ventilator-associated pneumonia. Recently, Perez-Köhler et al. developed a thermoresponsive rifampicin-loaded PNIPAM hyaluronan derivative hydrogel to coat polypropylene mesh materials.^31^ At 37 °C, an effective drug release showed strong antistaphylococcal activity both in vitro and in vivo. Taking into account the literature reports on polymer brushes capable of undergoing phase transitions in response to temperature changes, applying this concept to functionalize cotton for dressings could potentially enable the controlled release of drugs based on skin temperature fluctuations. At the same time, it may help minimize excessive moisture and exudate absorption, improving the dressing’s overall performance. Therefore, adhesion of the dressing to the wound or diseased area, because above the LCST, the polymer layer becomes hydrophobic. Thus, a thermosensitive polymer layer grafted onto cotton causes the material to transition from high water absorptive state below LCST to low absorptive state above LCST.^26^ A commonly used thermosensitive polymer for surface functionalization is PNIPAM; however, it has LCST of approximately 32 °C, which is below the physiological temperature. Additionally, PNIPAM has certain drawbacks, such as limited biocompatibility, which can vary depending on the concentration of residual monomer, the deposition method (when applied to a substrate), the cell line tested, and the presence of impurities.^32^ These limitations restrict its medical applications. As a result, polymers containing poly(ethylene glycol) (PEG) are being extensively studied. PEG-based polymers offer several advantages, including nontoxicity, biocompatibility, high solubility, and widespread clinical use. A key benefit of using polymers derived from ethylene glycol is the ability to fine-tune the LCST value within a broad range, from 26 to 90 °C. This can be achieved by selecting monomers with oligo(ethylene glycol) segments of varying lengths in their side chains, enabling the adjustment of their thermoresponsive properties to suit specific needs.^33−35^

In our study, we utilized SI-SARA ATRP with Cu^0^ serving as a supplemental activator and reducing agent to graft statistical copolymer brushes composed of DEGMA and OEGMA_500_ onto preactivated cotton. By adjusting the compositions of DEGMA and OEGMA_500_, we achieved precise control over the LCST behavior of the polymer layer. The LCST of the copolymers prepared in the reaction mixture, in the presence of the functionalized surface, was analyzed through transmittance measurements using ultraviolet/visible absorption spectroscopy (UV–vis) for aqueous solutions of P(DEGMA-*stat*-OEGMA). The thermoresponsive properties of the polymer layer grafted from the cotton surface were further examined by WCA measurements. Additionally, the temperature-dependent protein adsorption of polymer-functionalized cotton was investigated to evaluate the potential dressing’s adherence to wounds. The resulting materials were also tested for residual copper content and assessed for cytotoxicity.

## 2 Experimental Section

### 2.1 Materials

Polished silicon wafers were obtained from ON Semiconductor (Czech Republic) and cut into 1 × 2 cm^2^ pieces. Cotton, sourced from Technotex S.A., was prepared in 1 × 2 cm^2^ and 2 × 3 cm^2^ samples. Tetrahydrofuran (THF, ≥99.9%), *N,N*-dimethylformamide (DMF CHROMASOLV, ≥99.9%, GPC analysis), diethyl ether (C_4_H_10_O, ≥99.8%), ethanol (EtOH, ≥99.8%), hydrochloric acid (HCl, ≥37%, activation of copper wire before polymerization), and nitric acid (HNO_3_, ≥65%) were purchased from Honeywell Riedel-de Haën. Ethyl α-bromoisobutyrate (EBiB, 98%), copper(II) bromide (Cu^II^Br_2_, 99.9%), methanol (MeOH, 99.8%), toluene (C_7_H_8_, 99.8%), phosphate-buffered saline (PBS, pH 7.2–7.6), Dulbecco′s modified Eagle’s medium–high-glucose (DMEM), fetal bovine serum (FBS), penicillin-streptomycin, Cell Proliferation Kit I (MTT test), and hydrochloric acid (HCl, 37%, for ELISA tests) were purchased from Sigma-Aldrich. 3-(Trichlorosilyl)propyl 2-bromo-2-methylpropanoate (APTES-BiB, >95%) and 2-bromoisobutyryl bromide (BriBBr, >98%) were acquired from TCI Chemicals. Carbon disulfide (CS_2_, 99.9%) was purchased from Thermo Scientific. Triethylamine (TEA, 99%) and hydrogen peroxide (H_2_O_2_, 30%) were acquired from Chempur. Sodium hydroxide (NaOH, p. a.) was acquired from P.P.H. Stainlab. Albumin from bovine serum (BSA) labeled with Alexa Fluor 488 dye and rabbit antigoat IgG (H+L) HRP-labeled secondary antibody were acquired from Invitrogen, while Human primary dermal fibroblasts (HDFn, PCS-201-010) were acquired from ATCC, and 3,3′,5,5′-tetramethylbenzidine (TMB) was purchased from ImmunoChemistry Technologies. Proceeding substances were used without further purification. Tris(2-pyridylmethyl)amine (TPMA) was synthesized, as well as Cu^II^Br_2_/TPMA catalyst complex was prepared according to an earlier publication.^21,23^ DEGMA (95%, Sigma-Aldrich) and OEGMA_500_ (Sigma-Aldrich) were passed through a basic alumina column in order to remove the inhibitor.

### 2.2 Analyses

To study the kinetics of conducted reactions, proton nuclear magnetic spectroscopy (^1^H NMR) analyses were carried out in deuterated chloroform (chloroform-*d*), utilizing a Bruker Avance 500 MHz spectrometer (25 °C).

Molecular weights (MWs, *M_n_*) and molecular weight distributions (*M*_w_/*M_n_*) were determined by gel permeation chromatography (GPC) using a Shimadzu (Kyoto, Japan) modular system equipped with a CBM-40 system controller, SIL-20AHT automatic injector, the RID-20A differential refractive-index detector, and Repro-Gel 5 μm columns composed of precolumn and three Repro-Gel 5 μm columns (500, 10,000, and 1,00,000 Å). The temperature of the columns was maintained at 35 °C using a CTO-20A oven. The eluent was tetrahydrofuran (HPLC grade) and the flow rate was kept at 1 mL/min using an LC-40 pump. A molecular weight calibration curve was produced using commercial narrow molecular weight distribution polystyrene standards (PSS Polymer Standards Service).

The LCST of thermoresponsive copolymers was determined using a Hewlett-Packard Model HP-8453 diode array rapid scan spectrophotometer, which measures transmittance in the wavelength range from 200 to 1000 nm of a 1 g/mL polymer aqueous solution (LC-MS grade water) in a quartz cell with an optical length of 1 cm. Analyses were carried out for different sample temperatures in the range of 22–50 °C.

Ellipsometry measurements of polymer-modified silicon wafers (at incident angles of 60, 65, and 70°; spectral range: 350–1000 nm) were performed in air using a M200U spectroscopic ellipsometer (J. A. Wollam). The Cauchy model was applied to fit the obtained data, and the thickness values of polymer brushes were determined as the arithmetic mean of two measurements at different locations.

The chemical structure of cotton at every stage of functionalization was analyzed by attenuated total reflectance Fourier transform infrared spectroscopy (ATR-FTIR) using a Nicolet 6700 spectrophotometer (Thermo Scientific, Waltham, MA) in the range of 500–4000 cm^–1^.

Diffuse reflectance (DRS) spectra were recorded using a Shimadzu UV-3600 UV–vis-NIR spectrophotometer equipped with a 10 cm diameter integrating sphere.

Time-of-flight secondary ion mass spectrometry (ToF-SIMS) measurements were performed using the ToF-SIMS 5 (IONTOF GmbH, Münster, Germany) instrument, equipped with a 30 keV bismuth liquid metal ion gun. Bi_3_^+^ clusters were used as the primary ions with an ion dose density lower than 10^12^ ion/cm^2^ to ensure static mode conditions. To investigate the cotton surface chemistry at every stage of functionalization, negative ion spectra were acquired from at least four different and nonoverlapping spots (200 × 200 μm^2^ area each). During these measurements, a low-energy electron flood gun was used for charge compensation.

Scanning electron microscopy (SEM) micrographs were obtained on a Helios 5 Hydra DualBeam using immersion mode. The EDS analysis was performed using Apex software. The samples were sputtered with gold before imaging.

Thermogravimetric (TG) analysis was performed using a TGA 550 thermogravimetric analyzer from TA Instruments at a heating rate of 10 °C/min in nitrogen in a temperature range of 40–600 °C in open platinum pans, and the data was processed with TRIOS software. Differential scanning calorimetry (DSC) measurements were conducted by using DSC1 from Mettler Toledo. Measurements were performed under nitrogen (30 mL/min) using a heating and cooling rate of 10 K/min in the temperature range from −90 to 190 °C. Samples of ca. 4 mg were placed in sealed and pierced aluminum pans. Data analysis was performed by using STAR software.

Static contact angle experiments were performed by the sessile drop technique using a Kruss EasyDrop (DSA15) instrument in the Peltier temperature-controlled chamber. First, natural and modified cotton samples were placed in the chamber at temperatures set at 22, 30, 45, and 57 °C and left for at least 1 h to stabilize the temperature. Then, a drop of water at the approximate temperature of the surface was placed on the cotton sample and pictured with an LCD camera every 300 ms until thoroughly soaked. Then, the WCA were measured by using the software provided by the device producer.

### 2.3 General Procedure for Bromination of Silicon Wafers

Fragments of single-side polished silicon wafers (1 × 2 cm^2^) were sonicated in ethanol for 10 min and air-dried. To develop a layer of hydroxyl groups on the silicon wafers’ surface, they were put into UV ozone cleaner with a UV lamp with dominant wavelengths: 185 and 254 nm (Ossila, Sheffield, UK) for 30 min. Next, silicon wafers were placed polished side up into a conical flask (250 mL), submerged in a solution containing anhydrous toluene (30 mL), triethylamine (90 μL) and trichlorosilane initiator: (3-(trichlorosilyl)propyl 2-bromo-2-methylpropanoate) (15 μL), degassed under an argon stream for 40 min, and left for 24 h in an argon atmosphere. Thereafter they were sonicated for 5 min in anhydrous toluene and air-dried.

### 2.4 General Procedure for Chemical Activation and Bromination of Native Cotton

Six raw cotton samples (1 × 2 cm^2^ or 2 × 3 cm^2^) were placed in a mixture containing 42.0 mL of ethanol, 8.0 mL of carbon disulfide, and 0.70 g of sodium hydroxide; then, they were sonicated for 5 min and left submerged in the mixture for 2 h. Next, the cotton samples were cleaned by sonication in ethanol (1 × 5 min), water (4 × 5 min), ethanol (1 × 5 min), and tetrahydrofuran (1 × 5 min). Then, they were hanged on needles placed in rubber septa and inserted into a six-neck flask containing 40 mL of THF. The reaction space was degassed under an argon atmosphere for 40 min. Subsequently, 1.16 mL of TEA and 1.0 mL of BriBBr were slowly introduced into the flask. The cotton samples were then immersed in the reaction mixture, while the flask was placed in an ice bath for 2 h. The reaction was stopped after 22 h. The cotton samples were removed from the solution, cleaned by sonication in water (4 × 5 min) and ethanol (1 × 5 min), and then dried for 24 h in a vacuum desiccator at 60 °C.

### 2.5 General Procedure for Grafting P(DEGMA-*stat*-OEGMA) from Cotton via SI-SARA ATRP—Optimization of Reducing Agent Amount

The mixture of DMF (5.80 mL), DEGMA (4.70 mL, 25.4 mmol), OEGMA_500_ (1.30 mL, 2.83 mmol), Cu^II^/TPMA (170 μL of 0.05 M in DMF), and EBiB (6.90 or 20.8 μL, 0.05 or 0.14 mmol) was added to a Schlenk flask equipped with a magnetic stirrer bar. Subsequently, the silicon wafer (1 × 2 cm^2^) and cotton sample (1 × 2 cm^2^) were immersed in the reaction mixture. The reaction mixture was degassed for 15 min under an argon atmosphere. The polymerization was started by adding Cu^0^ wire (*l* = 6 or 12 cm^2^, *d* = 1 mm) to the reaction mixture. Copper wire was previously activated with HCl and placed in a rubber septum. Samples were withdrawn periodically to check *M_n_* and *M*_w_/*M_n_* of the polymers by GPC analysis and to follow monomer conversion using ^1^H NMR analysis. Before GPC analysis, the polymer samples were dissolved in THF (1 mL) + toluene (10 μL) as an external standard in the mobile phase and passed through a neutral aluminum oxide column equipped with a 0.22 μm syringe filter to remove the catalyst. The polymerization was stopped by opening the flask and exposing the catalyst to air.

### 2.6 General Procedure for Grafting P(DEGMA-*stat*-OEGMA) from Cotton via SI-SARA ATRP—Optimization of Monomers Molar Ratio

A couple of SARA ATRP reactions were carried out at different molar ratios of monomers: [DEGMA]/[OEGMA_500_] = 180/20 and [DEGMA]/[OEGMA_500_] = 184/16. The mixture of DMF (5.80 mL), DEGMA (4.70 or 4.90 mL, 25.5 or 26.7 mmol), OEGMA_500_ (1.30 or 1.10 mL, 2.83 or 2.32 mmol), Cu^II^/TPMA (170 or 174 μL of 0.05 M in DMF), and EBiB (20.8 or 21.3 μL, 0.14 or 0.15 mmol) was added to a Schlenk flask equipped with a magnetic stirrer bar. Further steps were performed as described above.

### 2.7 General Procedure for Grafting P(DEGMA-*stat*-OEGMA) from Cotton via SI-SARA ATRP—Upscaled Polymerization

The mixture of DMF (33.9 mL), DEGMA (28.7 mL, 0.156 mol), OEGMA_500_ (6.30 mL, 13.5 mmol), Cu^II^/TPMA (1.015 mL of 0.05 M in DMF), and EBiB (124.2 μL, 0.85 mmol) was added to a six-neck flask, already fitted with a magnetic stirrer bar. Further steps were performed as described above, noting that during the synthesis 6 cotton samples (1 × 2 cm^2^) were modified simultaneously and a 70 μm long Cu wire (*d* = 1 mm) was used.

### 2.8 Protein Adsorption Investigation

#### 2.8.1 Fluorescence

Adsorption of proteins to cotton fibers was studied by measuring the mean fluorescence of the probes. For this purpose, a solution of BSA labeled with Alexa Fluor 488 dye in PBS at a concentration of 1 μg/mL was prepared. The volume of 100 μL of the BSA solution was placed on top of unmodified and modified cotton fragments measuring 1 × 1 cm. The cotton was incubated with the BSA solution for 1 h at a temperature of 22, 37, or 45 °C. The cotton fragments were then rinsed in distilled water. The fluorescent images of the stained samples were collected with the same exposure time of 25 ms by using the Olympus IX51 microscope equipped with a 100W Mercury light source (Olympus U-LH100HG).

#### 2.8.2 ELISA

Adsorption of proteins to cotton fibers was examined by ELISA. For this purpose, a solution of rabbit antigoat IgG (H+L) HRP-labeled secondary antibody was prepared in PBS at a concentration of 1 μg/mL. Unmodified and modified cotton samples (1 x 1 cm^2^) were heated to 22, 37, and 45 °C. Then, 100 μL of the antibody solution was placed on samples, and they were incubated for 1 h in a humidified atmosphere at appropriate temperatures. After incubation, the cotton samples were rinsed in distilled water. To perform ELISA, 400 μL of TMB, the substrate for HRP, was added to the wells of a 24-well plate. Cotton fragments after incubation with the antibody were transferred to individual wells with TMB. As the control, modified and unmodified cotton samples not incubated with the antibody placed in the TMB solution were used. After approximately 2 min, when the solution changed color to blue, the samples were removed from the TMB solution. A 200 μL portion of 1 M HCl solution was added. Next, 100 μL of the total solution was transferred to five wells of a 96-well plate, and the absorbance of the solutions was measured at 560 nm with a scanning multiwell spectrophotometer (SPECTROstar Nano, BMG labtech).

### 2.9 Determination of Copper Concentration in Postreaction Cotton Sample by Atomic Absorption Spectrometry (AAS)

#### 2.9.1 Mineralization

Minor parts of the cotton sample (about 1 x 10 mm^2^) were mineralized in Teflon vessels using a microwave digestion system. To each sample 6 mL of fuming HNO_3_ and 2 mL of 30% H_2_O_2_ were added. The mineralization process was conducted at 200 °C within 10 min. The resulting solutions were diluted to 20 mL by using volumetric flasks.

#### 2.9.2 AAS Calibration Curve

The AAS calibration curve was achieved by using Cu^II^Br_2_ aqueous solutions acidified with HNO_3_. The concentrations of the standards were precisely prepared within the range of 0.5–1.5 mg of Cu^II^Br_2_/TPMA. Every point on the curve was the average of three individual measurements completed for each sample.

#### 2.9.3 AAS Measurements

Each of the samples obtained after the mineralization of the polymers was used directly for AAS analysis. Every result obtained was the average of five individual measurements performed for each sample. In the case of extensively high concentration of same samples, necessary dilution was carried out (that the measured value was within the reference curve range), which was taken into account in the final result.

### 2.10 Cytotoxicity Studies

#### 2.10.1 Cotton Incubation

Nonmodified and modified cotton samples (1 × 2 cm^2^) were incubated in 4 mL of Dulbecco’s modified Eagle’s medium (DMEM) with high glucose content, 10% FBS, and 1% penicillin-streptomycin solution at temperatures of 22, 37, or 45 °C. After 14 h, the cotton fragments were removed from the medium. Additionally, the pure medium was incubated at the same temperatures to be used in control experiments.

#### 2.10.2 Polymer Solution

To assess the influence of dissolved polymer P(DEGMA-*stat*-OEGMA) synthesized in the reaction mixture on cell morphology and viability, the solution of polymer in the complete medium was prepared in concentrations 1, 10, and 100 μg/mL.

#### 2.10.3 Cell Culture

HDFn cells were cultured in complete medium, which was DMEM with high glucose content, 10% FBS and 1% penicillin-streptomycin solution. Cells were cultured for 48 and 72 h in culture flasks at 37 °C in a humidified atmosphere in 5% CO_2_.

#### 2.10.4 MTT Test

To assess the viability of the cells, an MTT test was performed. The cells were cultured in the 96-well plate at a density of 5000 cells/cm^2^ in 100 μL of DMEM culture medium per well. After 1 day of cell culture, the medium was replaced with 100 μL of the polymer solutions, and the medium was incubated at different temperatures with cotton or the control medium. Additionally, cotton-incubated media were diluted 10 and 100 times and added to separate wells. The experiment was conducted in triplicate for each condition. The MTT test was performed 24 and 48 h after the medium change. MTT reagent solution was added to the culture medium in a volume of 10 μL. After 4 h of incubation with MTT at 37 °C, a solution of the solubilization buffer in a volume of 100 μL was added to each well. After 1 day of incubation at 37 °C in 5% CO_2_, solution absorbance at 560 nm was measured three times for a well with a scanning multiwell spectrophotometer (SPECTROstar Nano, BMG labtech). Cell morphology images were captured with a phase contrast microscope, an Olympus IX-51.

## 3 Results and Discussion

### 3.1 Functionalization of the Cotton

**Figure 1:**
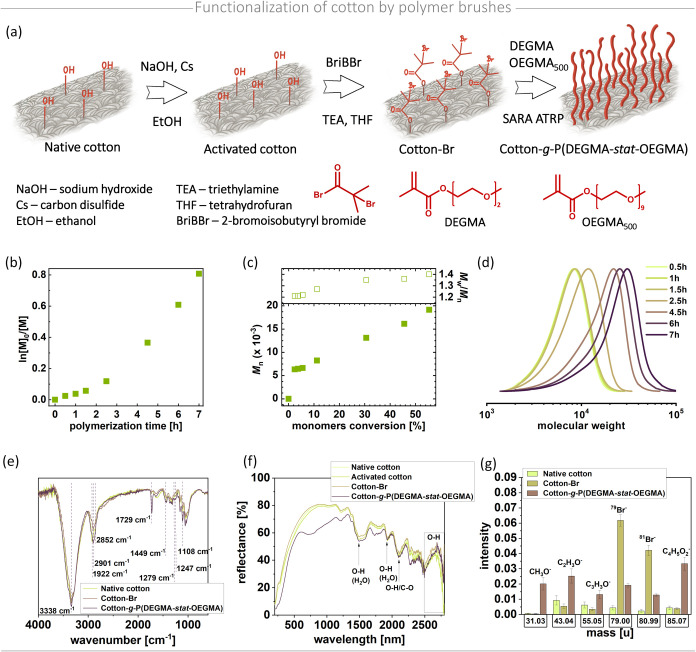
Functionalization of cotton surface by thermoresponsive polymer brushes. (a) Synthetic route. (b) Semilogarithmic plot of monomer conversion vs polymerization time. (c) *M_n_* and *M*_w_*/M_n_* vs monomer conversion. (d) GPC traces of copolymers composed of DEGMA and OEGMA_500_. (e) Normalized ATR FTIR spectra of native, brominated, and polymer-modified cotton. (f) DRS-UV–vis spectroscopy of cotton at all stages of functionalization. (g) Stacked column graphs presenting the intensity of negative ToF-SIMS secondary ions. Kinetics and chemical composition analysis is presented for the synthesis and synthesis products indicated in Table 1, entry 4.

Optimization reactions were performed to establish conditions for grafting P(DEGMA-*stat*-OEGMA_500_) brushes with precise structural and functional properties (Table 1, Figure 1a). All syntheses utilized EBiB as a sacrificial initiator to monitor polymerization kinetics in solution, with Cu^II^Br_2_ in its deactivated form as the catalyst and TPMA as the ligand. It is worth mentioning that the presence of a sacrificial initiator during surface functionalization helps maintain a controlled polymerization process by regulating the deactivation/activation equilibrium. Additionally, it reduces radical termination events at the surface, leading to more uniform and well-controlled polymer brush growth.^18,36^ Furthermore, a strong correlation between the molecular weights of these “free polymer chains” and the thickness of polymer brushes has been previously demonstrated.^37^ This suggests that the polymerization rate in solution is comparable to that of polymers grafted from all surfaces of the reaction mixture. To determine the thickness of the polymer brushes, silicon wafers (Si) were used as a reference surface. Since cotton is a porous and soft material, its roughness makes it unsuitable for direct thickness analysis using ellipsometry or atomic force microscopy (AFM). Initially, polymerization was conducted with varying amounts of initiator and Cu^0^ as both a supplemental activator and reducing agent, starting with a molar ratio of [DEGMA + OEGMA_500_]/[EBiB]/[ Cu^II^Br_2_/TPMA] = 600/1/0.18, along with a 6 cm copper wire (Table 1 , entry 1). However, this resulted in low monomer conversion and therefore a low-molecular-weight polymer (Figure S1). Under these conditions, modifying cotton would require excessively long reaction times, leading to prolonged exposure to organic solvents.

**Table 1:** Grafting P(DEGMA-*stat*-OEGMA) Brushes on the Cotton Surface via SI-SARA ATRP^a^

entry	*t* [h]	conv^b^ [%]	*k*_p_^app^^b^	*X*^c^ DEGMA [%]	*X*^c^ OEGMA [%]	*M*_*n*,theo_^d^ [×10^–3^]	*M*_*n*,app_^e^ [×10^–3^]	*M*_w_/*M*_*n*_^e^	*I*_eff_^f^ [%]	thickness^g^ [nm]	σ^h^ [nm^–2^]	LCST^i^ [°C]
1	21.0	5.67	0.016^j^	89.8	10.2	7,5	7,1	1.21	105	5 ± 1	0.46	*nd*

2	6.0	23.99	0.046	85.1	14.9	11,3	11,2	1.29	101	10 ± 1	0.56	43.5

3	6.9	70.34	0.166	75.3	24.7	37,3	19,9	1.37	188	22 ± 1	0.72	40.5

4	7.0	55.40	0.099	79.4	20.6	35,5	19,2	1.40	185	20 ± 1	0.68	39.5


a General reaction conditions: *T* = RT; argon atmosphere; [Monomer]_0_ = 50% v/v; *V*_*total*_ = 12 mL for entries 1–3 and V_*total*_ = 70 mL for entry 4; entry 1: [DEGMA]_0_ = 2.118 M, [OEGMA_500_]_0_ = 0.485 M, [DEGMA]/[OEGMA_500_]_0_/[EBiB]_0_/[Cu^II^Br_2_/TPMA]_0_ = 540/60/1/0.18; entry 2: [DEGMA]_0_ = 2.121 M, [OEGMA_500_]_0_ = 0.487 M, [DEGMA]/[OEGMA_500_]_0_/[EBiB]_0_/[Cu^II^Br_2_/TPMA]_0_ = 180/20/1/0.06; entries 3 and 4: [DEGMA]_0_ = 2.225 M, [OEGMA_500_]_0_ = 0.400 M, [DEGMA]/[OEGMA_500_]_0_/[EBiB]_0_/[Cu^II^Br_2_/TPMA]_0_ = 184/16/1/0.6. SARA ATRP with copper wire: *d* = 0.1 cm, *l* = 6 cm for entry 1; *d* = 0.1 cm, *l* = 12 cm for entries 2 and 3, and *d* = 0.1 cm, *l* = 70 cm for entry 4;

b Monomer conversion and apparent rate constant of propagation (*k*_p_^app^) were determined by ^1^H NMR spectroscopy;

c Molar fraction of DEGMA and OEGMA_500_ incorporated into copolymer calculated according to ^1^H NMR by comparing the integration of the vinyl proton −δ = 6.05–6.25 ppm (1 H), with the overall integration of the ethylene glycol protons −δ = 3.50–3.90 ppm (23 H) by subtracting the integration of the ethylene glycol protons in synthesized polymers characterized by chemical shifts in the same region, by comparison with the protons present in the first subunit of the PEG side chain of the monomers in the copolymer (δ = 4.05–4.25 ppm, 4 H);^43^

d *M*_*n*,theo_ = ((DP_theo_ · conversion · *x*_OEGMA500_/100 · *M*_OEGMA500_) + (DP_theo_ · conversion · *x*_DEGMA_/100 ·*M*_DEGMA_));

e Apparent *M*_*n*,app_ and *M*_w_/*M_n_* of polymers growing from the sacrificial initiator in the solution were determined by GPC, *M*_*n*,app_ is underestimated due to the molecular weight determination based on the linear PS calibration, which does not take into account the molecular weight of monomers side chains;

f Initiation efficiency, *I*_eff_ = (*M*_*n*,theo_/*M*_*n*,app_) · 100%;

g The thickness of the polymer brushes was determined on silicon wafers by ellipsometry and is the average of two measurements (the values refer to the total thickness of initiator layer and polymer layer);

h The grafting density (σ) was calculated by the following equation: σ = *N*_A_·h·ρ/M*_n_* assuming that the polymerization kinetics of SARA ATRP in solution and SI-SARA ATRP are comparable. *N*_A_ is the Avogadro constant, h is the brush thickness determined by ellipsometry measurement, ρ is bulk PDEGMA/POEGMA density (ρ = 1.08 g·cm^–3^,^44^ assumed the same value for both types of polymer), and *M_n_* is the number-average molecular weight value determined for polymers generated in the solution, σ values are overestimated due to the underestimation of *M*_n,app_;

i Lower critical solution temperature determined by UV–vis spectroscopy at polymer concentration of 1 mg mL^–1^ as the minimum value of the transmittance-temperature derivatives as a function of temperature (Figure S4);^35^

j Rate constant of propagation (*k*_p_^app^) was determined for kinetics samples up to 4 h of the reaction.

To address this, we increased the polymerization rate by raising the concentration of the ATRP initiator^38^ and enhancing the active surface area of Cu^0^ by using a 12 cm copper wire^39^ (Table 1, entry 2). This adjustment significantly accelerated polymerization, evidenced by a 3-fold increase in the apparent propagation rate constant. After only 6 h, a higher-molecular-weight polymer with a narrow molecular weight distribution was obtained, displaying a lower critical solution temperature (LCST) of 43.5 °C.

However, obtaining a polymer with an LCST of 43.5 °C is suboptimal for the intended use of modified cotton as a smart dressing material. Thermoresponsive drug carriers ideally release active substances at temperatures near physiological body temperature (approximately 36–40 °C). The LCST of statistical copolymers can be precisely tuned by adjusting the molar ratio of the comonomers. In this case, achieving a polymer with an LCST of 43.5 °C was accomplished by using monomers at a molar ratio of [DEGMA]/[OEGMA_500_] = 180/20 (Table 1, entry 2). To lower the phase transition temperature, the proportion of monomers with more ethylene glycol subunits should be decreased.^40^ Following this approach, a subsequent reaction with a molar ratio of [DEGMA]/[OEGMA_500_] = 184/16 yielded a polymer with an LCST of 40.5 °C (Table 1, entry 3), as anticipated, due to a decreased proportion of OEGMA_500_ in the resulting polymer chains. In general, the LCST values of the obtained polymers are comparable to those reported in the literature for the same monomer ratio.^41,42^ Minor differences can be attributed to variations in molecular weight, content of individual monomer subunits in the final composition, and the number of ethylene oxide units in the side chains of PEG-based monomers.^40,42^

The adjustment in monomer ratios also impacted polymerization kinetics: the reaction proceeded with a higher apparent propagation rate constant, yielding higher-molecular-weight chains at a significantly increased monomer conversion. To enable the simultaneous modification of multiple cotton samples and to observe the reaction system’s behavior at a larger scale, the reaction volume was increased to 70 mL from the initial 12 mL (Table 1, entry 4). The polymer produced at this scale had nearly identical molecular weights but showed a slightly broader molecular weight distribution (Figure 1b–d).

### 3.2 Physicochemical Properties of the Functionalized Cotton

The chemical composition of the prepared polymer-functionalized cotton was verified by using ATR-FTIR and DRS-UV–vis spectroscopy, ToF-SIMS, and SEM coupled with EDS spectroscopy. Additionally, SEM enabled the visualization of the surface morphology.

Successful grafting of polymer chains from the cotton structure was confirmed by comparing the FTIR spectra of cotton at each step of the functionalization (Figure 1e). The bromination step is challenging to confirm clearly when comparing native and brominated cotton, due to the low concentration of bromine relative to the overall material. A slight difference in the spectra of polymer-functionalized samples compared to other samples is observed around the bands at 1247 cm^–1^ and 1279 cm^–1^. These bands form a characteristic doublet associated with the crystalline phase and amorphous phase of PEO, attributed to antisymmetric and symmetric twisting vibrations of −CH_2_ groups.^45^ Similarly, a band at 1449 cm^–1^, corresponding to weak C–H bending vibrations of the −CH_2_ groups in poly(ethylene glycol)-based monomers, is also observed.^46^ Additionally, a more intense band at 1108 cm^–1^ is evident, corresponding to the antisymmetric stretching vibration of C–O–C.^45^ The signal at 1108 cm^–1^ also aligns with the C–O–C stretching and C–C breathing modes of cellulose ring, making it visible in the spectra of native and brominated cotton surfaces as well.^47^ A distinct difference is visible between the spectra of polymer-modified and native or brominated cotton at 1729 cm^–1^, attributed to the C=O stretching vibration from the carbonyl group.^48^ Moreover, unmodified cotton shows antisymmetric and symmetric stretching vibrations of C–H at 2852 and 2922 cm^–1^, respectively.^49^ However, in the spectrum of cotton functionalized with bromide and polymer, only the band at 2901 cm^–1^ is present, corresponding to C–H asymmetric stretching vibrations.^50^ The band exhibited at ca. 3400 cm^–1^ is attributed to the O–H stretching vibrations. At first, the intensity of this peak decreases after chemical activation and bromination of cotton. This decrease originates from removing saturated C:16 and C:18 hydroxy-fatty acids – the main ingredients of cutin, which are removed by NaOH treatment.^51^ After cotton was grafted with polymer brushes, the signal from the O–H groups increased. This phenomenon is probably caused by enhanced moisture in the fiber’s core, which could increase by absorbing water molecules from the air before the polymerization step.

Further evaluation of cotton modification was employed by DRS-UV–vis spectroscopy (Figure 1f). The spectrum of native cotton revealed characteristic features consistent with existing literature findings.^52^ The bands at 1450 and 1900 nm are attributed to hydroxyl (−OH) groups, related to groups exposed on the fiber surface and adsorbed water molecules. In the range of 2500–2750 nm, a broad reflectance band corresponding to the O–H group was also present. Moreover, a distinct band at 2150 nm was also observed, associated with the presence of O–H and C–O bonds.^53^ The observed trends in intensity changes corresponding to the stages of modification follow FTIR analysis. The main change in the reflectance spectra is observed in the range of 600–1000 nm, where the strong decrease of the reflectance values observed for polymer-grafted cotton originates from light absorption by P(DEGMA-*stat*-OEGMA) molecules. This observation confirms the effective coverage of the cotton fiber with polymer brushes.

ToF-SIMS was performed to confirm the successful modification by identifying elemental and molecular entities on the modified surface. To effectively represent the specific signals for negative ions recorded for unmodified cotton (Cotton), initiator-modified cotton (Cotton-Br), and polymer-modified cotton, stacked column graphs were prepared (Figure 1g). The successful bromination of the cotton surface is indicated by two prominent peaks corresponding to the stable bromine isotopes (^79^Br^–^ and ^81^Br^–^, *m*/*z* = 79.00 and 80.99, respectively).^24,54^ Following the grafting of polymer chains, the intensity of these bromine signals significantly decreases, confirming the success of the grafting process. The presence of polymer brushes is further validated by the appearance of CH_3_O^–^, C_2_H_3_O^–^, C_3_H_3_O^–^, and C_4_H_5_O_2_^–^ negative ion signals, which originate from the copolymer’s side chains.^55^ The presented results confirmed the efficient functionalization of cotton by polymer brushes.

**Figure 2:**
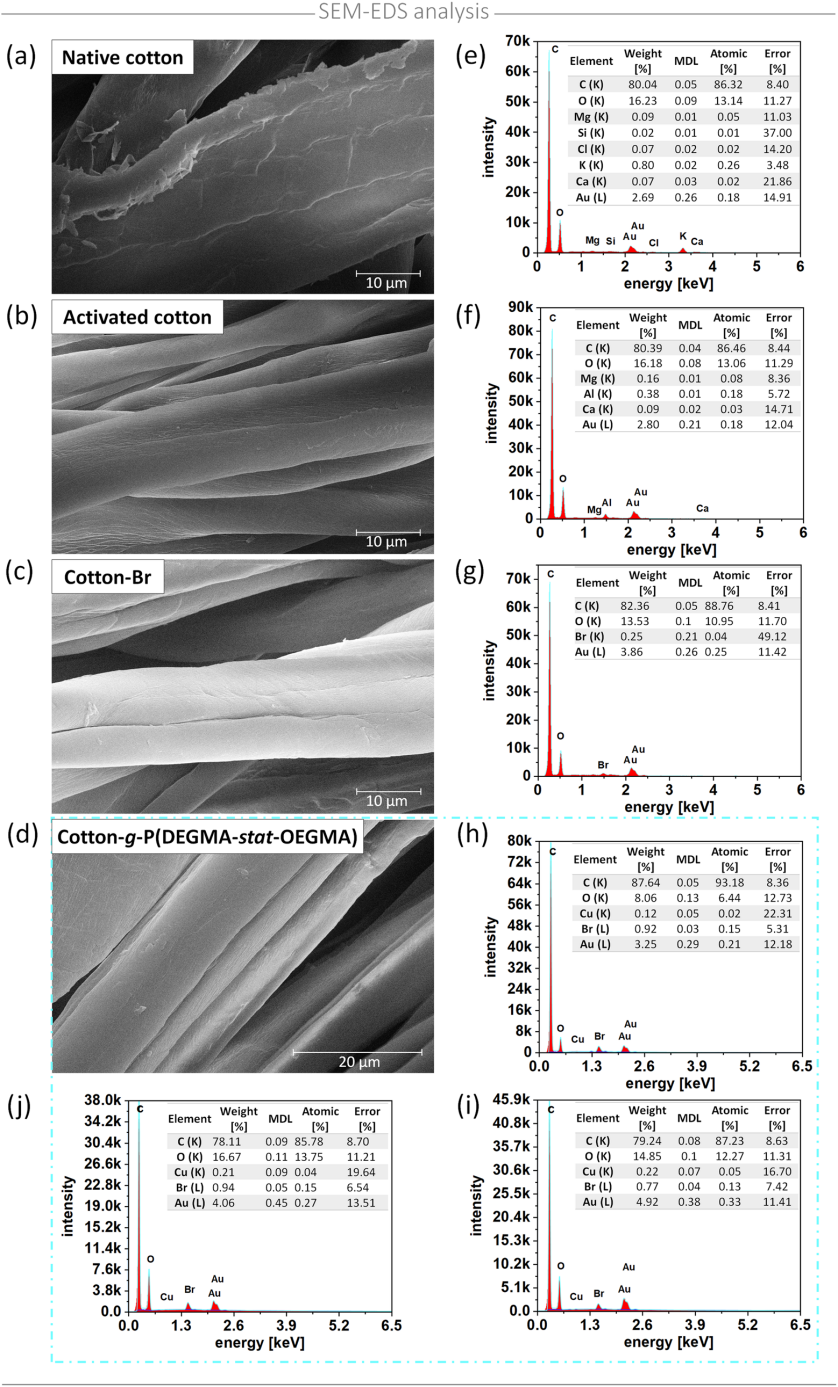
SEM micrographs and corresponding EDS spectrum of cotton surface: (a, e) native, (b, f) activated, (c, g) brominated, and (d, h–j) P(DEGMA-*stat*-OEGMA)-modified cotton. For polymer-modified cotton, EDS measurements were performed at three sample locations to access homogeneity of the polymer layer.

Analysis of the SEM-EDS results (Figure 2) revealed the presence of carbon (C) and oxygen (O) in all spectra, corresponding to the components of the cotton fibers. In the spectrum of cotton functionalized with the ATRP initiator, a signal from bromine (Br) was also detected. After the subsequent modification of the cotton with polymers, the bromine signal remained visible, indicating the preservation of halogen at the terminal end of the polymer chains. To verify the uniformity of the polymer coating on the cotton surface, EDS spectra were recorded at different locations on the sample. The intensity of the bromine signal was consistent across all measurements, confirming the homogeneity of the polymer coating. SEM also provided insights into the morphological features of the functionalized surfaces (Figure 2). Imaging revealed that unmodified cotton fibers exhibit a typical rough structure similar to that of native cotton. The activation step removed the hydrophobic cuticle and uncovered the smooth surface of the primary cell wall, leading to a decrease in the fiber diameter from 20–40 μm to below 15 μm.^56^ The bromination of the fibers did not additionally affect the surface morphology. However, after the grafting P(DEGMA-*stat*-OEGMA), the characteristic pattern of parallel wrinkles appeared, which was also observed for cotton modified via ARGET ATRP.^57^

These observed morphological changes combined with elemental analysis are consistent with the results obtained from ATR-FTIR and ToF-SIMS analyses. This correlation confirms the successful grafting of P(DEGMA-*stat*-OEGMA) brushes onto the cotton surface.

### 3.3 Thermal Properties of the Functionalized Cotton

The effect of cotton functionalization on thermal stability was analyzed using thermogravimetric analysis (Table S1, Figure S5). Unmodified and activated cotton demonstrated slightly higher thermal stability compared with brominated or polymer-modified cotton. For these samples, the temperature of the maximum weight loss rate (DTG_max_) appeared at 368 and 369 °C, respectively, which is associated with the degradation of cellulose fibers.^58^ The lowest decomposition temperature was observed for the sample with an immobilized ATRP initiator. This can be attributed to the thermal dissociation of small-molecule compounds from the surface, which requires less energy compared with the decomposition of biopolymer structures. Thus, it can be concluded that surface modification of materials intended for high thermal resistance is more effective when macromolecular compounds are used. Despite the differences observed, it is important to note that the surface modification with polymers compared to low-molecular-weight compounds such as ATRP initiator increased the thermal resistance of the brominated cotton material. This enhancement is particularly significant for the storage and handling of polymer-modified cotton at elevated temperatures, such as during drying processes or exposure to sunlight. Additionally, DSC measurements were conducted. The results revealed no significant differences between the cotton samples at any modification stage. This is likely due to the low concentration of the modifier within the structure of the functionalized surface, which falls below the instrument’s detection limit (see details in Section S4. *Thermal properties of functionalized cotton* in the Supporting Information).

### 3.4 Analysis of Thermoresponsive Properties of the Functionalized Cotton Surface by Measuring the Water Contact Angle

**Figure 3:**
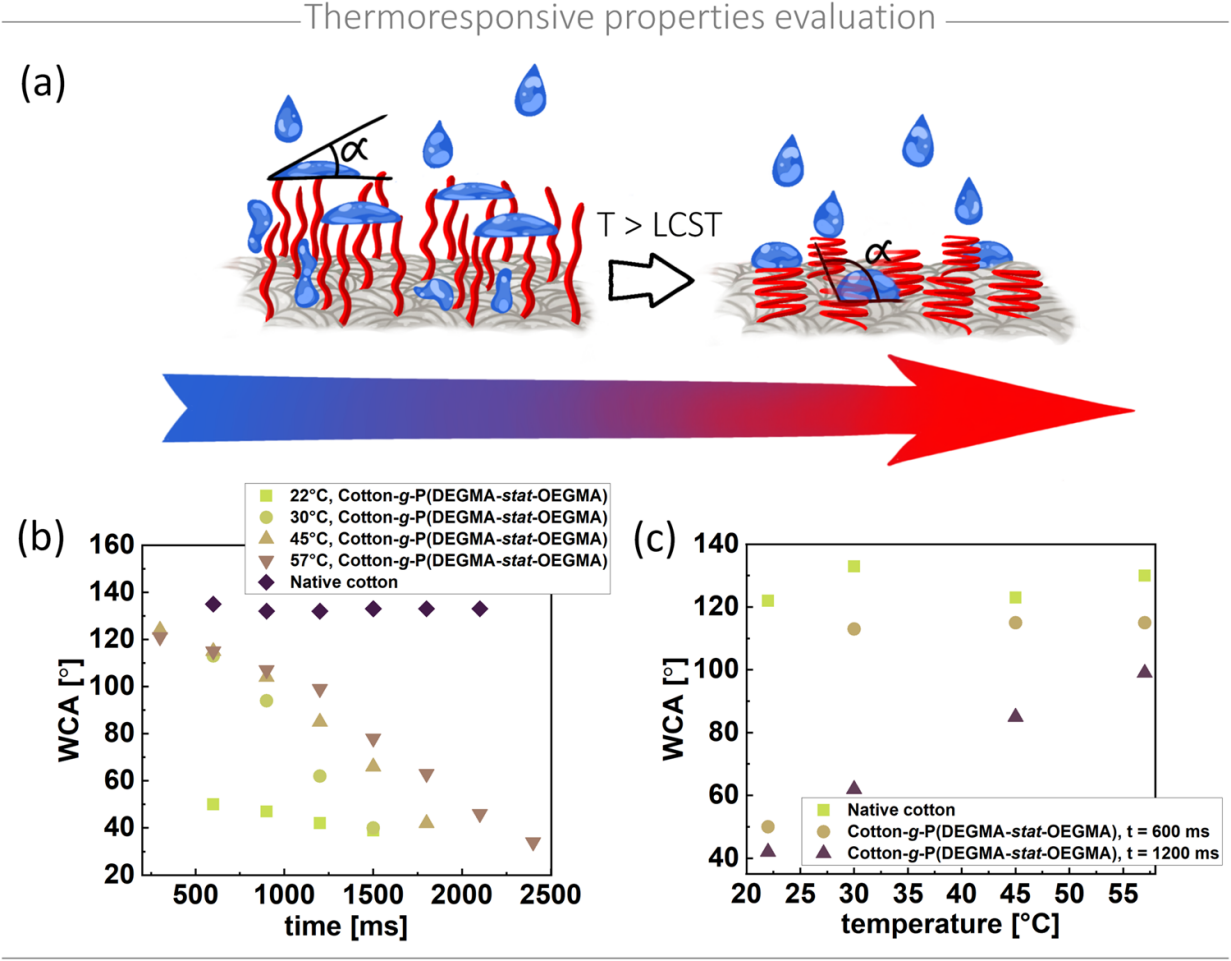
Evaluation of thermoresponsive behavior of polymer-modified cotton by WCA measurements at various temperatures: (a) Schematic representation of the transition of PEG-based polymer brushes grafted from cotton, shifting from hydrophilic to hydrophobic in response to temperature changes. (b) Effect of temperature on the WCA of native and polymer-functionalized cotton over the measurement time. (c) WCA of native and polymer-modified cotton measured at 600 and 1200 ms as a function of temperature.

The prepared thermoresponsive cotton-polymer material has the potential to release active substances from its brush structure in response to the local environmental temperature. This feature makes it particularly suitable for applications such as treating burns or localized inflammation, where the affected area is characterized by an elevated skin temperature. To assess its potential application in the aforementioned context, the thermoresponsive behavior of the polymer brushes grafted onto the cotton surface was evaluated using WCA measurements conducted across a temperature range of 22–57 °C (Figure 3). At room temperature (22 °C), native cotton fabric exhibited hydrophobic characteristics with a WCA of approximately 130°. This behavior is likely attributed to the high grammage (550 g/m^2^) and dense weave, which were selected to prevent material disintegration during the modification process because the samples, ranging in size from 1 × 1 cm^2^ to 2 × 3 cm^2^ were functionalized. Moreover, functionalizing this type of cotton enabled the assessment of the thermoresponsive behavior of the prepared material while minimizing the influence of the inherently hydrophilic cotton, which would absorb aqueous solutions regardless of polymer grafting. This feature is typical for lightweight, i.e., low, grammage cotton, which is usually used to produce dressings because it ensures that the dressing remains breathable, therefore does not exert unnecessary pressure on the wound or surrounding tissue, and is easy to handle and conform to the contours of the body.^59^ In contrast, polymer-functionalized cotton exhibited significantly altered behavior, with the WCA decreasing to approximately 40° and showing complete water absorption within 1500 ms. It is a result of the hydrophilic characteristic of P(DEGMA-*stat*-OEGMA) polymer chains below the LCST. Therefore, it confirms the efficient functionalization of cotton. After increasing the local temperature of WCA measurement, the functionalized cotton exhibited thermoresponsive characteristics. In general, as the measurement temperature increased, the surface of the functionalized cotton exhibited progressively more hydrophobic behavior. At 30 °C, the WCA increased to 113° within 600 ms, with complete soaking observed after 1500 ms. At 45 °C, the droplet persisted slightly longer, displaying higher contact angles at each measurement interval. At 57 °C, the surface demonstrated significantly enhanced hydrophobic characteristics, with the droplet remaining on the surface even after 2400 ms.

The diminishing difference in wettability between the hydrophobic surface of unmodified cotton and the thermosensitive material as the temperature increases is clearly illustrated in Figure 3c. At room temperature, the unmodified and modified materials exhibit distinctly different behaviors. However, at 55 °C, the reduction in the contact angle of the functionalized surface over time during exposure to a water droplet is significantly smaller. This observation suggests the presence of a covalently bonded hydrophobic polymer coating with thermosensitive properties.

The complete absorption of the water droplet into the polymer-functionalized cotton at elevated temperatures over time was likely due to droplet cooling under ambient conditions. Initially, the droplet was at the same temperature as that of the substrate. However, during its transfer and after contact with the surface, it probably rapidly cooled to ambient temperature, affecting the material’s wetting behavior and resulting in increased absorption.

The demonstrated differences in the properties of polymer-functionalized cotton suggest its potential for the controlled release of active substances from wound dressings.^60^ Below the LCST P(DEGMA-*stat*-OEGMA) polymer brushes are hydrated and swollen and adopt an extended conformation due to strong interactions between the polymer chains and water molecules. The active substance, which may be embedded within the brush matrix or adsorbed onto the polymer chains, is retained within the swollen polymer network. Above LCST, the polymer brushes undergo a phase transition. They become dehydrated and collapse into a dense, compact state as the hydrogen bonding between the polymer and water is disrupted. This change in conformation alters the diffusivity and/or interaction of the active substance with the polymer, leading to its release.^44^ Due to the hydrophilic nature of polymers below their LCST, this type of macromolecules favors encapsulation of hydrophilic active substances typically used in burns treatment e.g., low-molecular-weight compounds e.g., vitamins^61^ or antibiotics.^28,62^ Brushes retain hydrophilic substances due to favorable hydrogen bonding and hydrophilic interactions. Above LCST, the polymer chains collapse, the brush becomes more hydrophobic, and the water content decreases. Hydrophilic substances are expelled due to the loss of favorable interactions and shrinking of the hydrophilic environment.

Considering the results for prepared polymer-functionalized cotton, at a room temperature of 22 °C, the dressing can safely store the active compounds without triggering their release. However, upon contact with the skin – where the normal temperature is approximately 32–34 °C, and inflamed or burned areas may exhibit temperatures several degrees higher^63^ – there is a potential gradual release of active substances. This release becomes increasingly effective as the temperature of the affected skin rises.

Additionally, a more hydrophobic surface of polymer-modified cotton at temperatures above the LCST compared to usually used cotton dressings^59^ reduces the absorption of moisture and exudate. This, in turn, minimizes the adhesion of the dressing to the wound or affected area, thereby enhancing both the effectiveness and comfort of the dressing’s use.

### 3.5 Protein Absorption Tests

**Figure 4:**
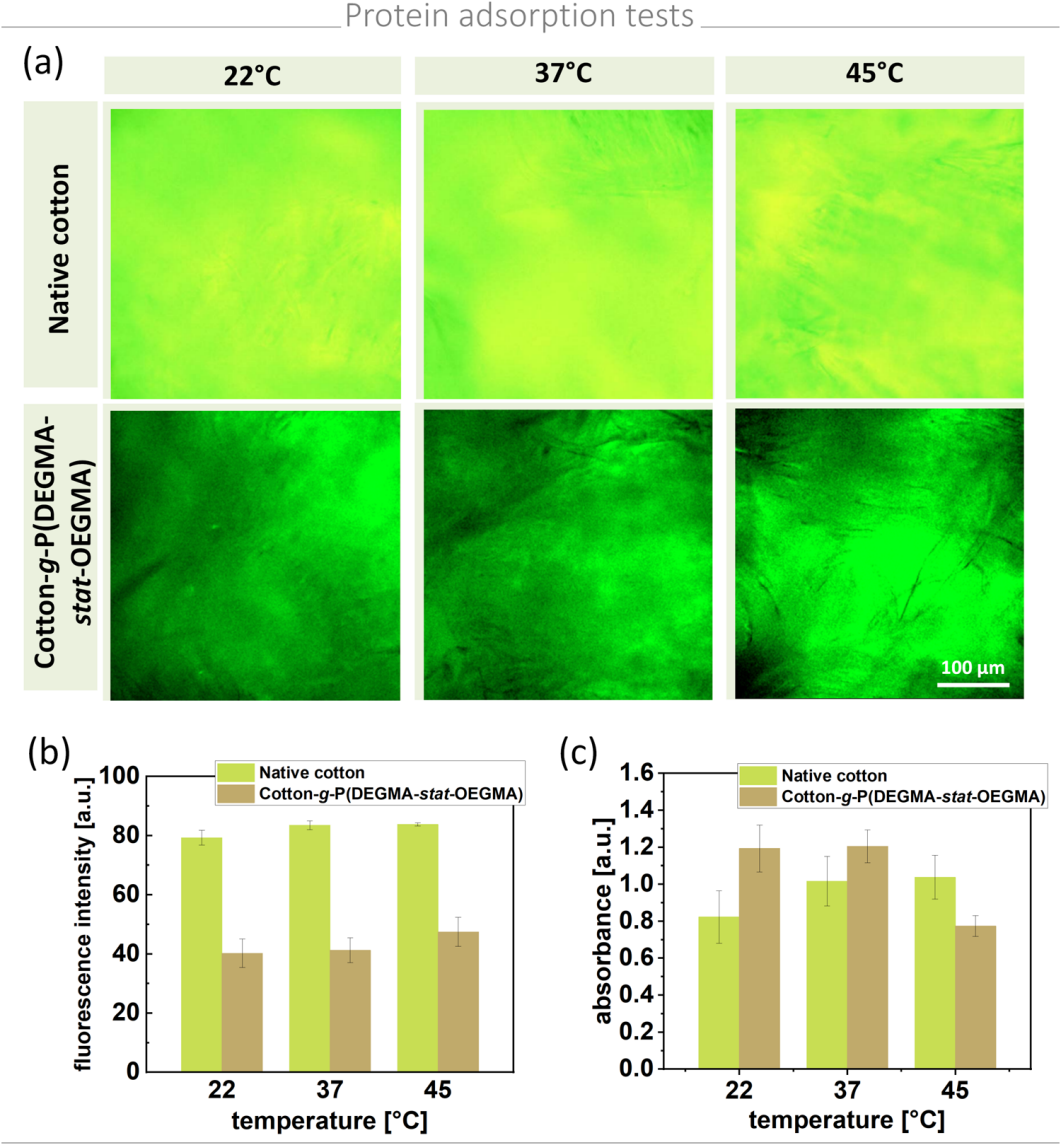
(a) Fluorescence images and (b) intensity corresponding to the amount of protein applied to cotton vs temperature. (c) Adsorption of proteins to cotton fibers examined by ELISA.

The P(DEGMA-*stat*-OEGMA) layer exhibits antifouling properties,^21,64,65^ prompting protein adsorption tests on unmodified and modified cotton materials. Two methods were employed: fluorescence measurements to analyze protein adsorption to the cotton surface (Figure 4a,b) and an enzyme-linked immunosorbent assay (ELISA) test to assess protein absorption throughout its volume (Figure 4c).

Fluorescence measurements revealed that, while temperature changes did not significantly affect protein adsorption, native cotton strongly adsorbed proteins on its surface. In contrast, protein adsorption was reduced by half on polymer-modified cotton, confirming the surface’s antifouling properties. Even at elevated temperatures, when the PEG-based polymer layer becomes more hydrophobic, protein adsorption on the modified surface was significantly lower compared to that of the unmodified material. Observed changes may be related to the wettability of the surface, which governs nonspecific protein adsorption. Proteins adsorb to hydrophobic surfaces, whereas hydrophilic surfaces are considered antifouling due to the formation of a hydration layer that reduces protein–surface interactions, leading to low or negligible protein adsorption.

However, note that, in the case of cotton samples, a drop of protein solution soaks into the material. The hydrophilic surface of the polymer-grafted cotton facilitates transport of protein solution into the material’s volume, whereas the hydrophobic surface of unmodified cotton impedes such penetration, which leads to a significantly higher content of protein solution in functionalized samples compared to the unmodified material. The ELISA test provides information from the entire volume of the cotton sample, so it is sensitive to proteins adsorbed not only to the cotton surface but also to the fibers in the interior of the sample. Therefore, the amount of proteins detected by ELISA is higher for modified hydrophilic samples. Consequently, the two measurement methods yielded opposite results. Additionally, the ELISA test revealed the thermosensitive nature of the polymer-modified cotton with significant changes observed at 45 °C. Above the LCST, the polymer-modified material becomes more hydrophobic. Although hydrophobic materials generally exhibit higher protein absorption,^66^ the results showed reduced protein uptake at elevated temperatures. This phenomenon supports the hypothesis that surface characteristics influence protein transport into the material’s interior. As the surface becomes more hydrophobic, the absorption of the protein solution into the material is hindered, leading to decreased protein absorption above the LCST. This characteristic is advantageous for wound dressings as it minimizes adhesion to the wound site, thereby reducing discomfort, wound damage, and complications during dressing changes.

In summary, protein adsorption analyses highlighted distinct differences between unmodified and modified materials as well as the thermosensitive nature of polymer brushes covalently grafted to cotton. These findings underscore the functional advantages of polymer-modified cotton, particularly in applications requiring antifouling and thermoresponsive properties.

### 3.6 Cytotoxicity Analysis

To evaluate the potential cytotoxicity of the polymers attached to the cotton surface and fabricated coatings, two types of tests were conducted by using dermal fibroblast cultures. To determine the cytotoxicity of copolymers grown in the reaction mixture, they were added to the culture medium and their impact on cell viability was analyzed using MTT colorimetric assays, which measure the conversion of MTT into formazan crystals by living cells, reflecting their mitochondrial activity.^67^ The results showed no statistically significant differences in cell viability reduction between polymer concentrations of 1 and 100 μM, even after 48 h of incubation (Figures S8 and S9).

**Figure 5:**
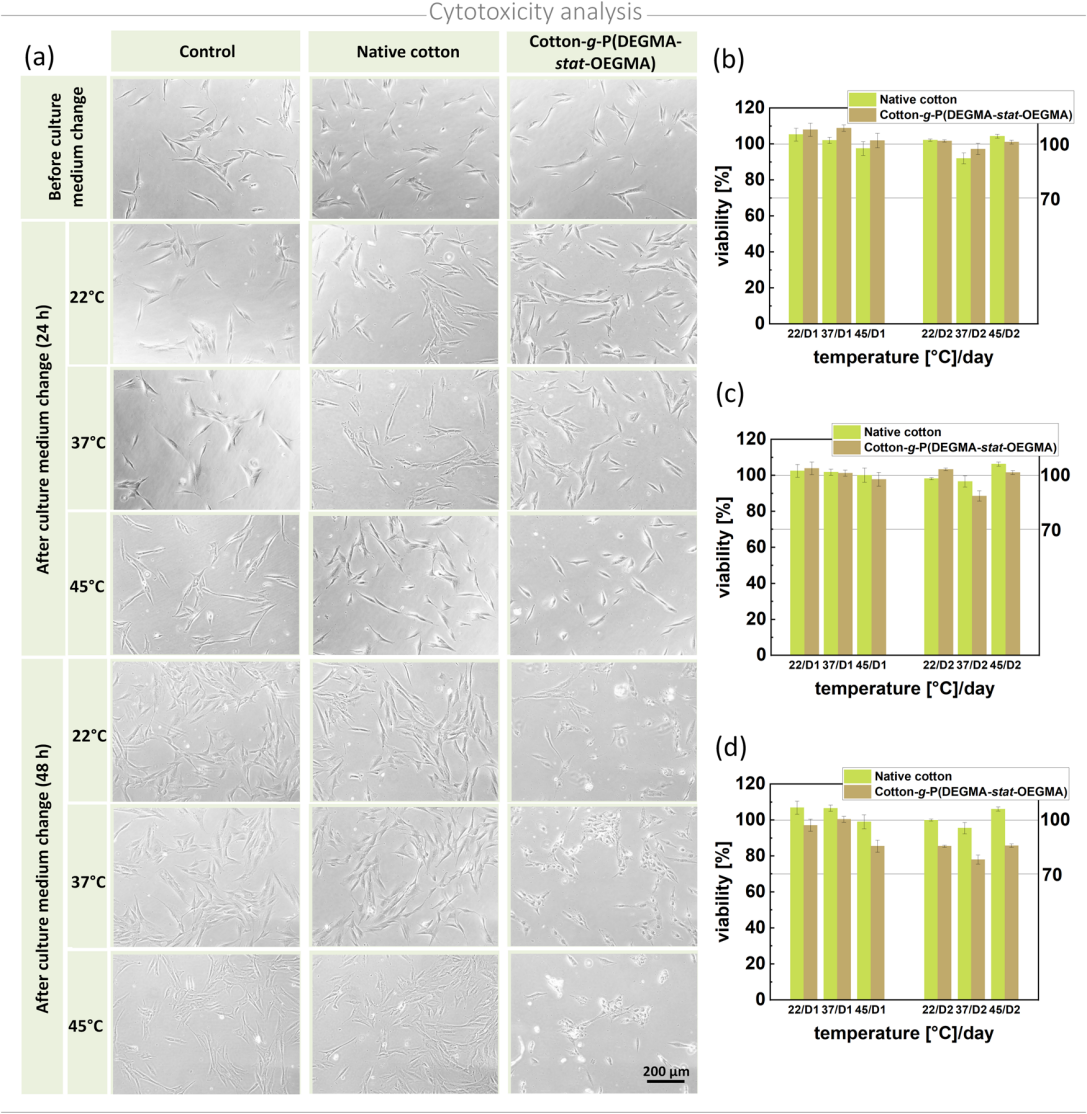
(a) Morphology, visualized with a phase contrast microscope, and (b–d) viability, assessed with a scanning multiwell spectrophotometer of the dermal fibroblasts cultured in (d) obtained extract and its (c) 1:10 and (b) 1:100 dilution.

For polymer-modified cotton, the impact of the thermoresponsive coating on cell viability at different temperatures was examined (Figure 5). For this purpose, both unmodified and functionalized cotton samples were incubated in standard culture medium at different temperatures. Then, the standard medium in cell culture was exchanged by obtained extracts and their 1:10 and 1:100 dilutions, and cell viability was traced using the MTT test. It was observed that extracts obtained from unmodified cotton did not affect the viability of cells. In turn, the cytotoxicity of polymer-modified cotton on dermal fibroblasts depended on the extract concentration and incubation time. The cytotoxic effect diminished significantly with a 10-fold dilution of the extract (Figure 5c) and was no longer detectable with a 100-fold dilution (Figure 5b). Regarding incubation time, a noticeable effect was observed primarily at higher extract concentrations and longer incubation periods (Figure 5d). After 48 h, some cells displayed changes in shape and signs of shrinkage (Figure 5a). However, overall analysis of the data indicates that cell viability across all extract concentrations remained above 70% after 48 h, suggesting that the materials are noncytotoxic.^68^ These findings affirm the safety of modified cotton and its potential for biomedical applications.

### 3.7 Copper Content in Polymer-Functionalized Cotton

Given the observed decrease in the viability of dermal fibroblasts incubated with polymer-modified cotton, alongside the absence of cytotoxicity from the polymer itself (Figure S11) and the unmodified cotton, the potential influence of residual copper (Cu^0^) in the material as a catalyst residue and reducing agent was investigated by determining the copper content in the purified polymer-modified cotton by AAS. After synthesis, the material underwent multiple purification cycles using sonication in THF and ethanol. Initially, after the first purification cycle, approximately 0.5 wt % of Cu^0^ remained in the modified cotton. Following five purification cycles, the copper concentration was reduced to approximately 0.08 wt %, and after eight cycles, the copper content decreased further but plateaued. This suggests that additional purification cycles would not significantly lower the copper content further. This is likely due to the penetration of Cu^0^ into the cotton fibers, making it difficult to fully remove, a challenge exacerbated by the presence of the covalently attached polymer layer. It is hypothesized that residual Cu^0^ contributes to the observed reduction in dermal fibroblast viability. However, as the cell viability remains above 70%, the material can still be classified as noncytotoxic, making it a viable candidate for biomedical applications. Moreover, the copper content in the material may be especially beneficial in application for chronic wound healing.^69^ It is worth noting that copper has antimicrobial properties, which are attributed to its ability to release copper ions. These ions damage the cell membranes and other structures of microbes, ultimately leading to their destruction.^70^ Therefore, copper residues in the final polymer-functionalized cotton can enhance its effectiveness for such applications.

## 4 Conclusions

Summarizing, this paper introduces a method for precisely tuning the thermoresponsive properties of polymer-modified cotton through the polymerization of DEGMA and OEGMA_500_ using the SI-SARA ATRP technique. By adjusting the molar ratio of comonomers in a controlled polymerization process, the LCST value of the statistical copolymers can be finely tailored. Spectroscopic, spectrometric, and microscopic analyses confirmed the successful grafting of P(DEGMA-*stat*-OEGMA) brushes onto the cotton surface. Water contact angle measurements revealed temperature-dependent differences in the properties of the polymer-functionalized cotton. At a room temperature of 22 °C, the functionalized cotton exhibited hydrophilic characteristics, making it suitable for safely storing active compounds without initiating their release. As the temperature increased, starting at approximately 30 °C, the polymer-modified surface transitioned to progressively more hydrophobic behavior. At normal body temperatures of around 32–34 °C, or in inflamed or burned areas where temperatures may be several degrees higher, it is expected that the dressing facilitated a gradual release of active substances. The functionalized surface also demonstrated reduced absorption of moisture and exudate at temperatures above the LCST compared to usually used cotton dressings, thereby minimizing adhesion of the dressing to the wound or affected area. This property enhances both the effectiveness and comfort of the dressing, making it a promising solution for advanced wound care applications.

Protein adsorption on the surface of the modified material remained relatively constant, despite temperature changes. However, when analyzing protein adsorption throughout the entire volume of the modified material, an unexpected decrease in protein absorption was observed at elevated temperatures. This finding supports the hypothesis that surface characteristics significantly influence protein transport into the material’s interior. As the surface becomes more hydrophobic at temperatures above the LCST, the absorption of the protein solution into the material is hindered, leading to reduced protein absorption. This property is particularly advantageous for wound dressings as it minimizes the absorption of moisture and exudate. By preventing excessive adhesion to the wound site, this feature reduces the risk of secondary injury during dressing changes, thereby lowering patient discomfort, minimizing wound damage, and reducing complications associated with dressing changes.

Moreover, cytotoxicity tests indicate that cell viability in the presence of polymer-functionalized cotton remained above 70% after 48 h, confirming that the material is noncytotoxic. These findings validate the safety of modified cotton and highlight its potential for biomedical applications.

This study presents an innovative approach to designing advanced wound dressings with key features, such as low adherence, reduced absorption of moisture and exudate, and the potential ability to manage the controlled release of antibiotics or drugs in response to environmental changes.

## Supporting Information Available

Polymerization kinetics investigation, spectroscopic analysis of purified polymer, thermoresponsive characteristics of polymers, thermal properties of functionalized cotton, cytotoxicity of polymers, and AAS results (PDF)
