# Supplementary material for: Functionalization of Cotton by Thermoresponsive Polymer Brushes for Potential Use as Smart Dressings
Source: ACS Appl Polym Mater. 2025 Apr 18;7(9):5646–60. doi: 10.1021/acsapm.5c00534 (PMC12070369; doi:10.1021/acsapm.5c00534)
Supplement: Supplementary file 1 — ap5c00534_si_001.pdf [file ap5c00534_si_001.pdf]

Supporting Information for

Functionalization of cotton by thermoresponsive polymer brushes for potential use as smart dressings

Izabela Zaborniak,[,1,2] Michał Sroka,[1] Kamil Wilk,[1] Anna Cieślik,[3,4] Joanna Raczkowska,[4] Kaja Spilarewicz,[5] Natalia Janiszewska,[3,4] Kamil Awsiuk,[4] Karol Wolski,[5] Kinga Pielichowska,[6] Paweł Błoniarz,[1] Katarzyna Kisiel,[1,7] Magdalena Bednarenko,[1] Krzysztof Matyjaszewski[2] and Paweł Chmielarz[*,1,2]*

[1]Department of Physical Chemistry, Faculty of Chemistry, Rzeszow University of Technology, al. Powstańców Warszawy 6, 35-959 Rzeszów, Poland

[2]Department of Chemistry, Carnegie Mellon University, 4400 Fifth Ave., Pittsburgh, PA 15213, United States

[3]Doctoral School of Exact and Natural Sciences, Jagiellonian University, Łojasiewicza 11, 30-348 Kraków, Poland

---

[*] Corresponding author. E-mail: p_chmiel@prz.edu.pl; i.zaborniak@prz.edu.pl

[4]Faculty of Physics, Astronomy and Applied Computer Science, M. Smoluchowski Institute of Physics, Jagiellonian University, Łojasiewicza 11, 30-348 Kraków, Poland

[5]Faculty of Chemistry, Jagiellonian University, Gronostajowa 2, 30–387 Kraków, Poland

[6]Department of Glass Technology and Amorphous Coatings, Faculty of Materials Science and Ceramics, AGH University of Krakow, Al. Mickiewicza 30, 30-059 Kraków, Poland

[7] Doctoral School of the Rzeszow University of Technology, Rzeszow University of Technology, al. Powstańców Warszawy 8, 35-959 Rzeszów, Poland

Contents

S1. Kinetics studies on grafting [DEGMA-*stat*-OEGMA$_{500}$] brushes on the cotton surface via SI-SARA ATRP – optimization of reaction conditions 4

S2. Spectroscopic analysis of purified polymer 6

S3. Dependence of lower critical solution temperature of P(DEGMA-*stat*-OEGMA) on the molar ratio of the monomers 7

S4. Thermal properties of functionalized cotton 9

S5. Cytotoxicity of polymers 11

S6. Determination of copper concentration in the post-reaction cotton sample by atomic absorption spectrometry (AAS) 12

S1. Kinetics studies on grafting [DEGMA-*stat*-OEGMA$_{500}$] brushes on the cotton surface via SI-SARA ATRP – optimization of reaction conditions

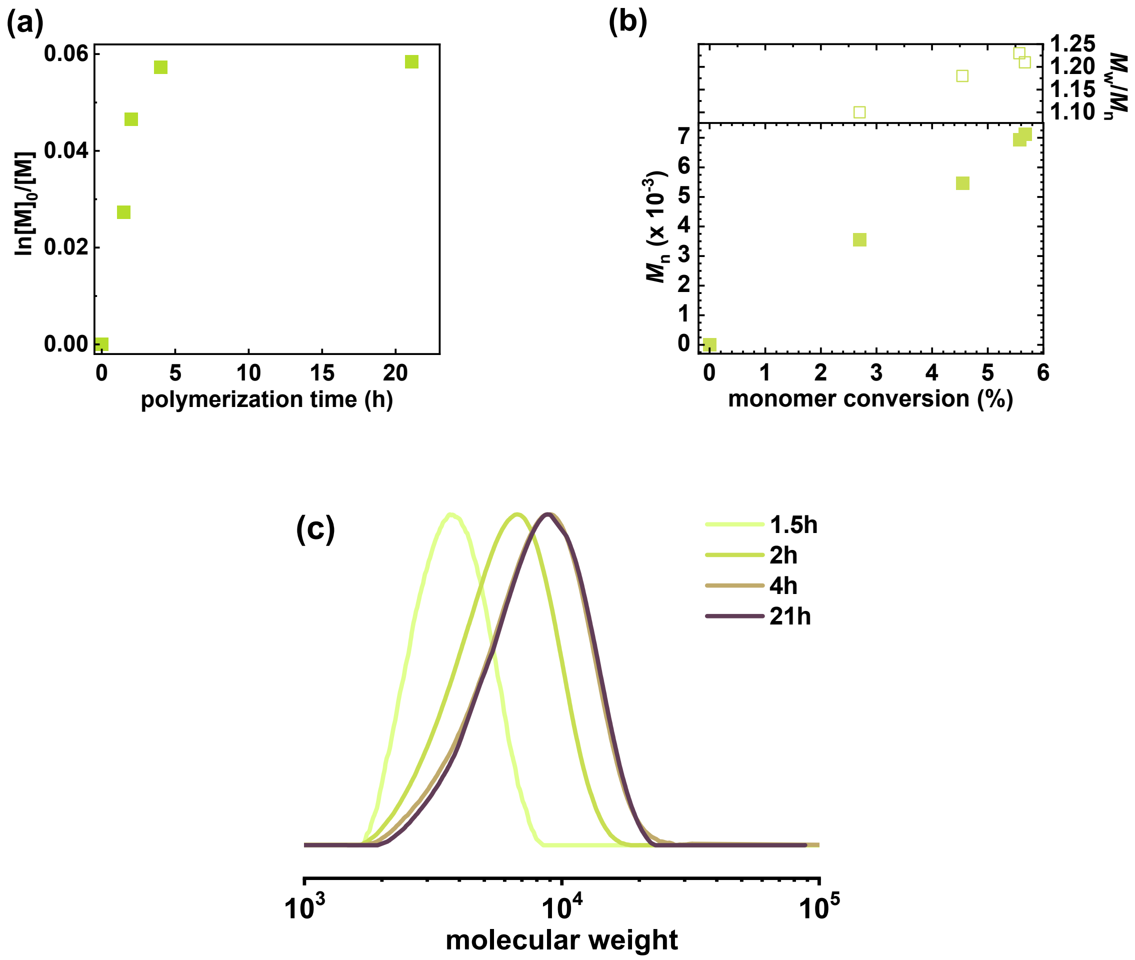

Figure S1. a) Semilogarithmic plot of DEGMA and OEGMA$_{500}$ conversion vs. polymerization time; (b) M_n and M_w/M_n vs. DEGMA and OEGMA$_{500}$ conversion; (c) GPC traces of DEGMA and OEGMA$_{500}$ polymerization (**Table 1**, entry 1).

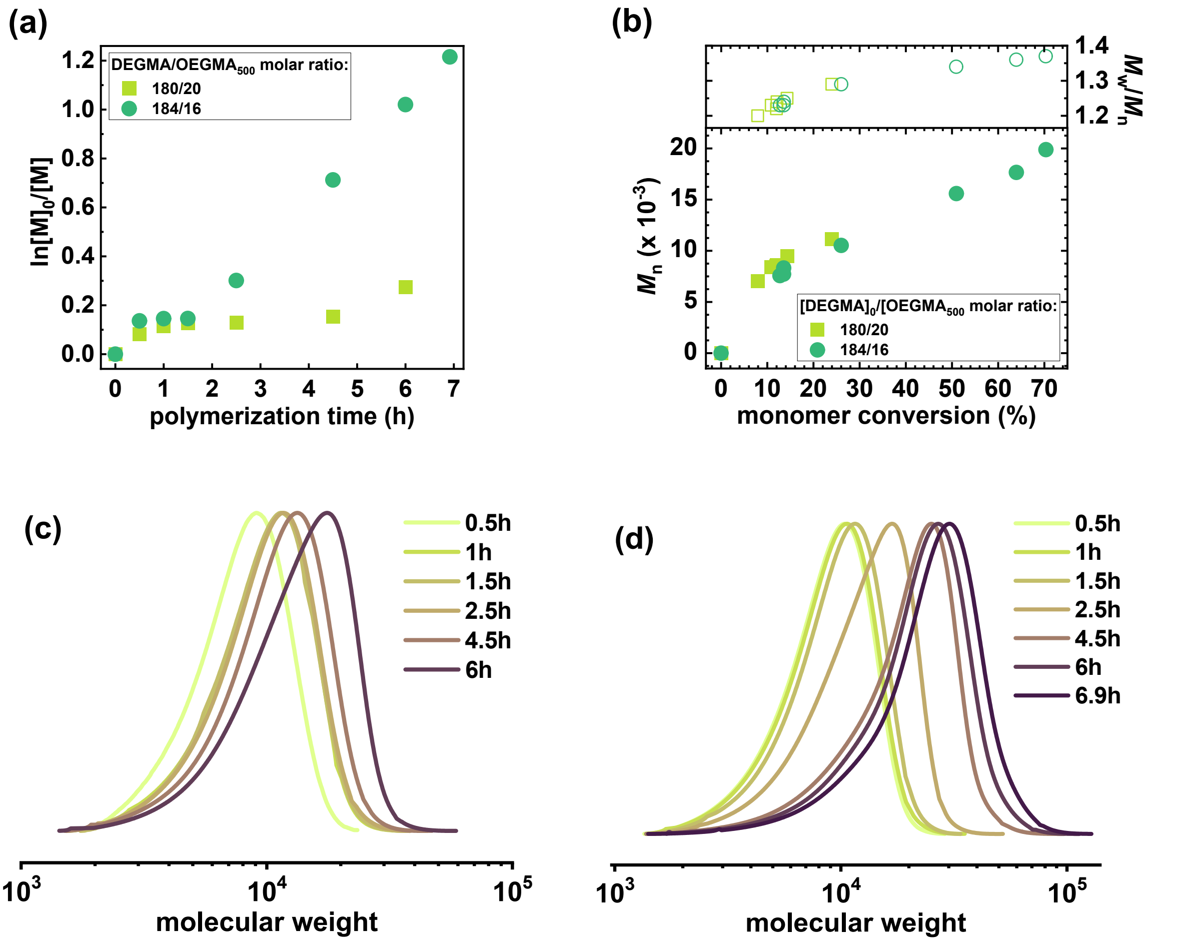

Figure S2. a) Semilogarithmic plot of DEGMA and $OEGMA_{500}$ conversion vs. polymerization time (Table 1, entries 1 – 2); (b) M_n and M_w/M_n vs. DEGMA and $OEGMA_{500}$ conversion (Table 1, entries 1 – 2); (c) GPC traces of DEGMA and $OEGMA_{500}$ polymerization (Table 1, entry 1); (d) GPC traces of DEGMA and $OEGMA_{500}$ polymerization (Table 1, entry 2).

S2. Spectroscopic analysis of purified polymer

The chemical shifts identified in the ^{1}H NMR spectrum, shown in **Figure S3** clearly evidenced PDEGMA-*stat*-POEGMA as follows: δ (ppm) = 0.60–1.18 (3H, CH_3–, α), 1.62–2.17 (2H, –CH_2–, β), 3.28–3.45 (3H, –CH_3, c), 3.48–3.75 (8H, –CH_2–, a + b) and 3.90–4.43 (2H, –CH_2–, a in the first segment).[1]

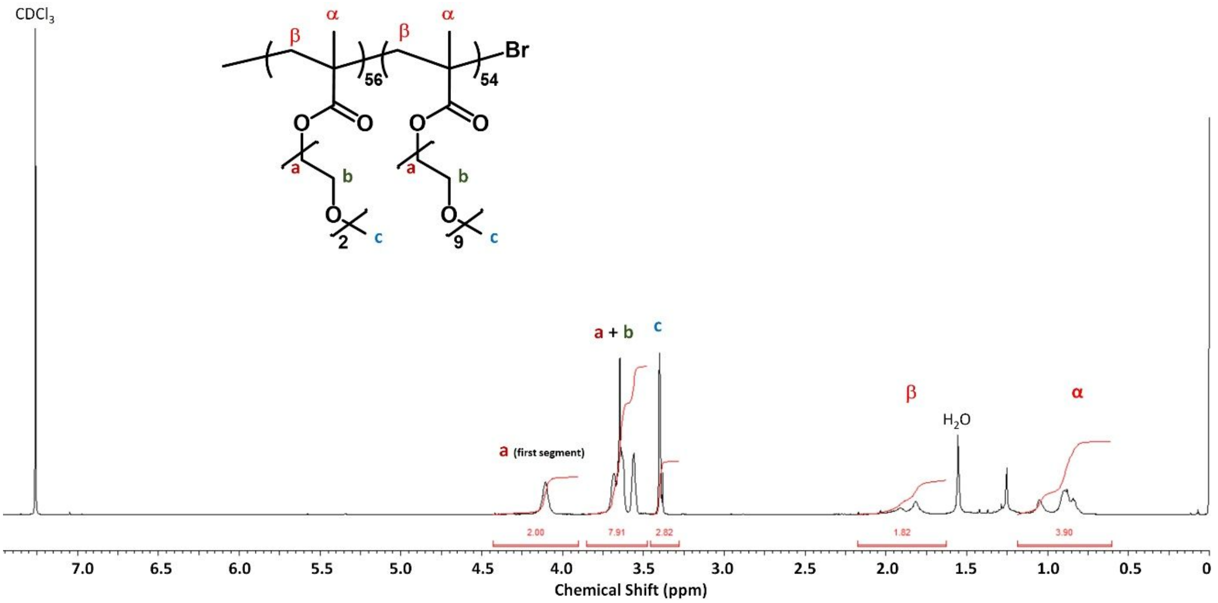

Figure S3. ^{1}H NMR spectrum of PDEGMA-*stat*-POEGMA (M_n = 19 200, M_w/ M_n = 1.40) in $CDCl_3$, obtained *via* SARA ATRP (**Table1, entry 4**).

S3. Dependence of lower critical solution temperature of P(DEGMA-*stat*-OEGMA) on the molar ratio of the monomers

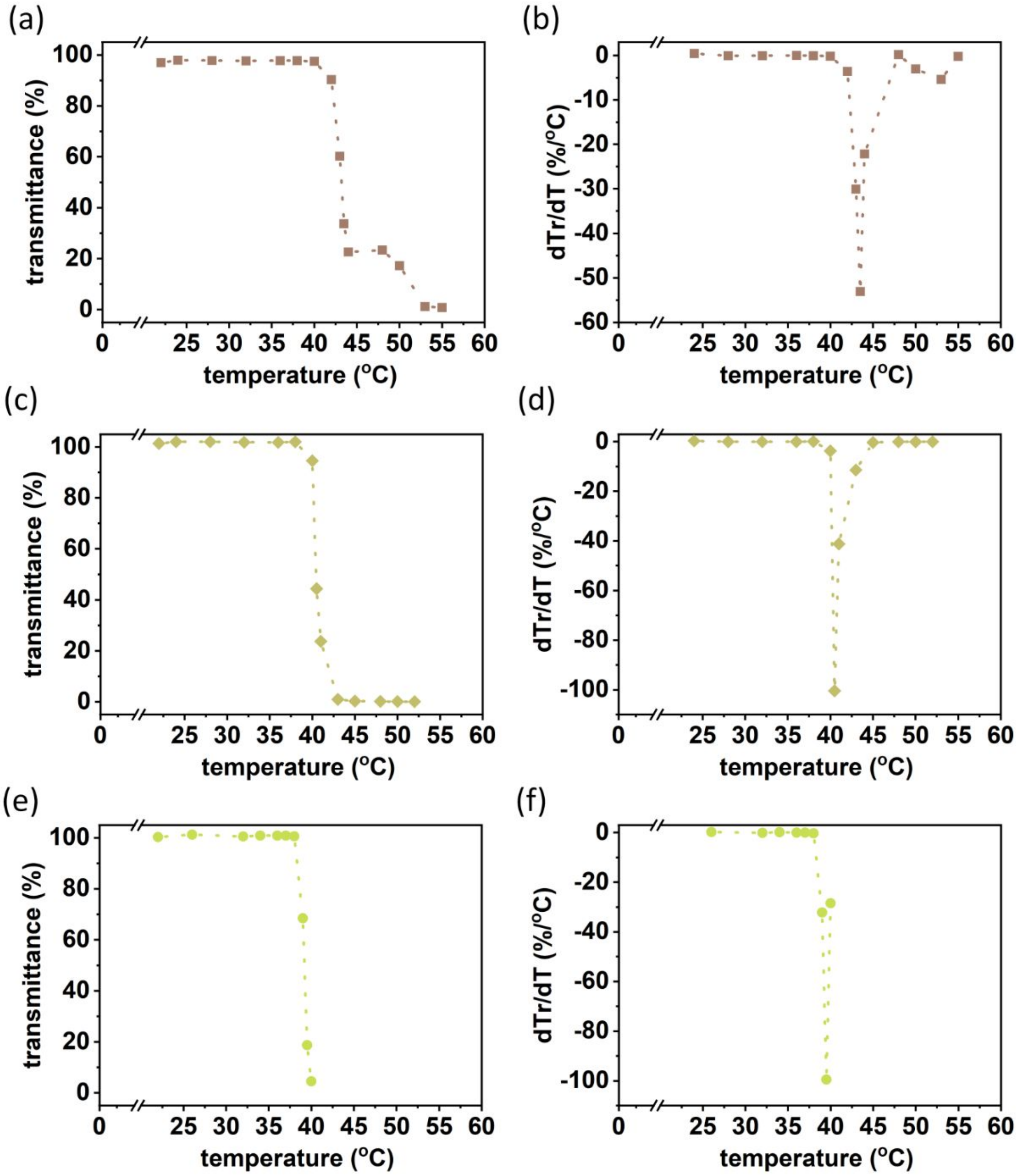

Figure S4. Transmittance vs temperature and derivatives of the transmittance-temperature data for P(DEGMA-*stat*-OEGMA) aqueous solutions prepared using [DEGMA]/[$OEGMA_{500}$] molar ratio of (a, b) 180/20 (**Table 1**, entry 2), and 184/16 at total reaction mixture volume of (c, d) 12 mL (**Table 1**, entry 3) and (e, f) 70 mL (**Table 1**, entry 4).

S4. Thermal properties of functionalized cotton

Table S1. Thermal stability of cotton at every stage of functionalization.

Sample	Temperature of the % mass loss [°C]					DTG_{max}
	$T_{1\%}$	$T_{3\%}$	$T_{5\%}$	$T_{10\%}$	$T_{50\%}$	[°C]
Cotton	275	310	324	339	365	368
Activated cotton	280	315	327	340	366	369
Cotton-Br	239	260	268	281	338	347
Cotton-*g*-P(DEGMA-*stat*-OEGMA)	251	283	297	314	351	355

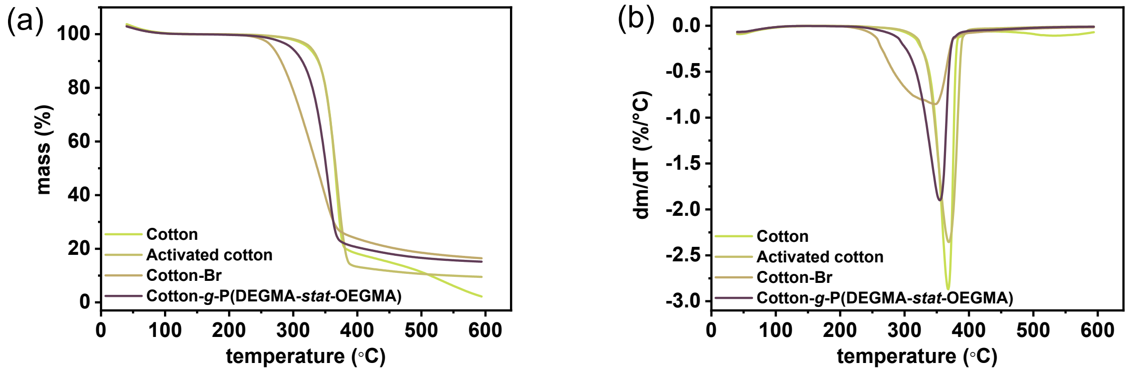

Figure S5. (a) TG and (b) DTG curves of cotton at every stage of functionalization.

The differential scanning calorimetry (DSC) curves of polymer-functionalized cotton over a wide temperature range exhibit a broad endothermic peak at lower temperatures, between 79˚C and 82˚C (**Figure S6**). This peak is attributed to the desorption of water molecules adsorbed onto the fiber surface.[2] Notably, no glass transition was detected for the P(DEGMA-stat-OEGMA) polymer chains grafted onto the cotton surface, which would typically be observed between -35˚C and -59˚C, corresponding to the glass transition temperatures (T_g) of pure PDEGMA and POEGMA, respectively.[3] This absence is likely due to the low concentration of

the polymer modifier on the functionalized surface, rendering it undetectable by the instrument. Additionally, there were no significant differences observed in the DSC curves for cotton samples at any stage of modification (**Figure S7**).

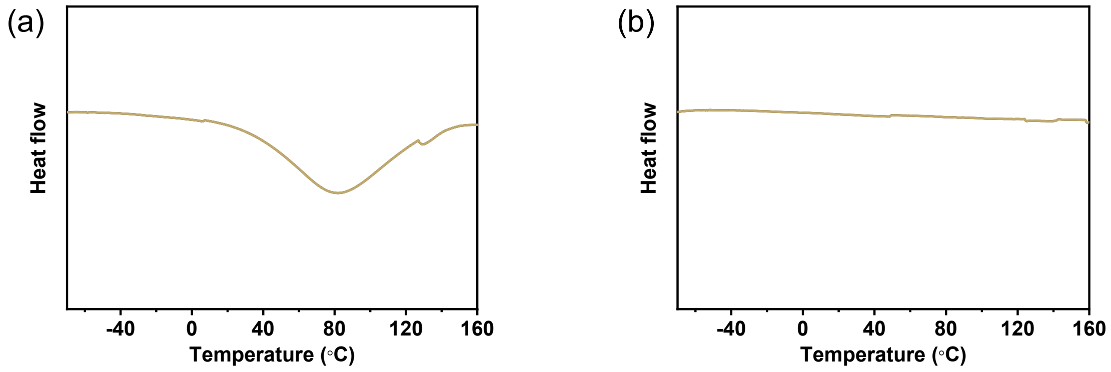

Figure S6. DSC curves of polymer-functionalized cotton at over a wide range of temperatures: (a) first heating, (b) second heating.

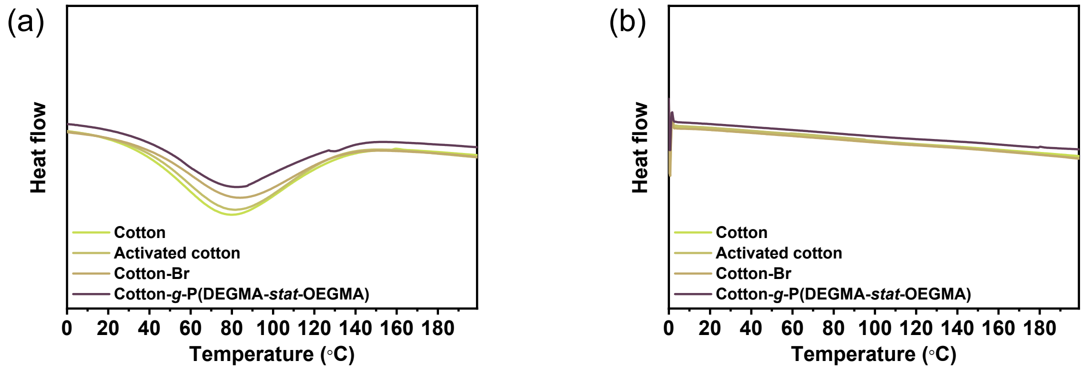

Figure S7. DSC curves of cotton at every stage of functionalization: (a) first heating, (b) second heating.

S5. Cytotoxicity of polymers

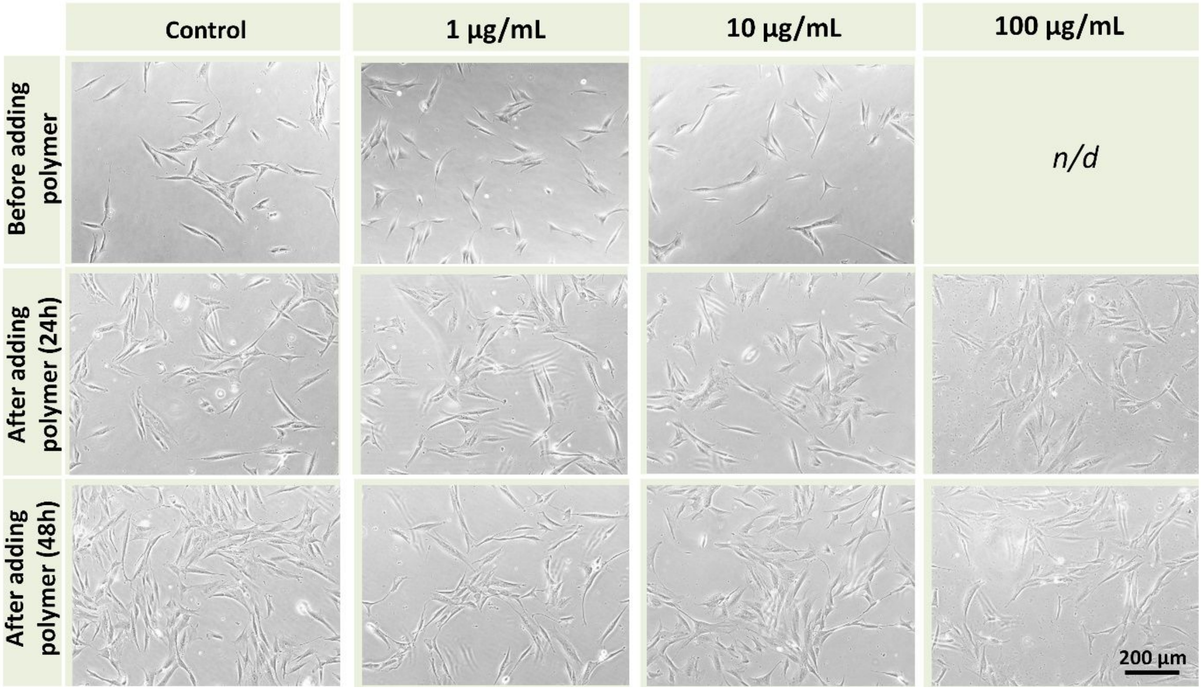

Figure S8. The morphology of the dermal fibroblast colony incubated in the polymer solution.

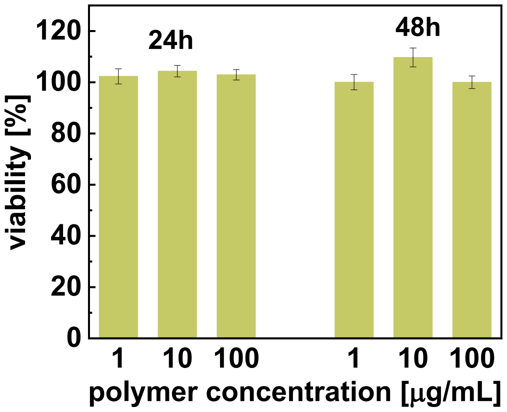

Figure S9. Dermal fibroblasts viability vs. polymer concentration.

S6. Determination of copper concentration in the post-reaction cotton sample by atomic absorption spectrometry (AAS)

The AAS technique is a commonly employed method for quantifying metal residues in polymer material samples. In this context, it was utilized to assess the copper concentration remaining in the polymer-polymer modified cotton after purification by washing the cotton in THF and ethanol in ultrasonic bath – one washing cycle is sonication 4 times THF and ones in ethanol. The washed functionalized cotton underwent decomposition in a drying oven and subsequent mineralization within a microwave digestion system. This process left behind only the copper content, which was atomized in a flame during the measurement. The concentration was determined through the use of the equation illustrated in **Figure S10**:

$$c_{Cu} = \frac{A}{0{,}05278}$$

where A – absorbance [-], c_{Cu} – copper concentration [mg/L].

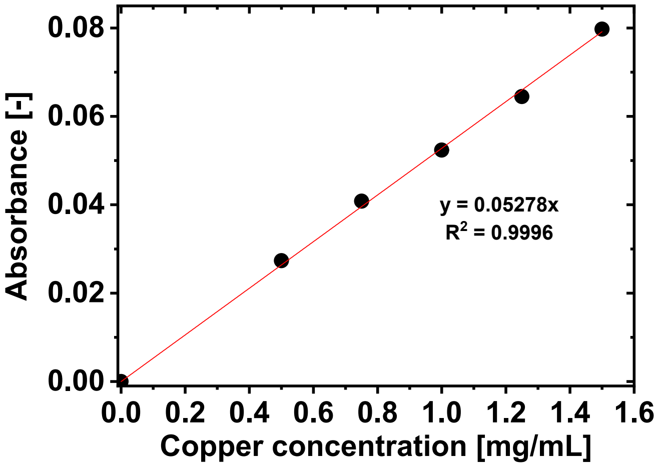

Figure S10. The calibration curve established by analyzing $Cu^{II}Br_2$ reference solutions in distilled water by AAS with a copper hollow cathode lamp, measurements were taken at a wavelength of 324.8 nm, and a flame atomizer.

The copper concentration in the functionalized cotton after purification determined by AAS based on the calibration curve was summarized in **Figure S11**.

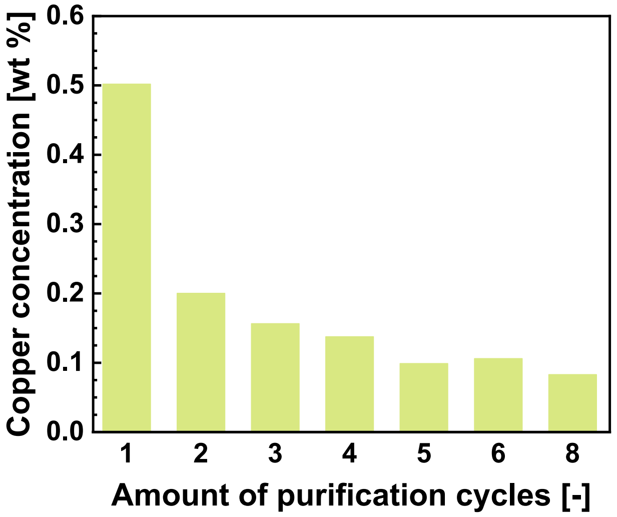

Figure S11. Copper concentration in the post reaction cotton sample after each purification cycle – one purification cycle includes washing the cotton in THF (4x) and ethanol (1x) in ultrasonic bath.

REFERENCES

[1] Iborra, A., Díaz, G., López, D., Giussi, J. M., Azzaroni, O., Copolymer based on lauryl methacrylate and poly(ethylene glycol) methyl ether methacrylate as amphiphilic macrosurfactant: Synthesis, characterization and their application as dispersing agent for carbon nanotubes, Eur. Polym. J. 87 (2017) 308-317. https://doi.org/10.1016/j.eurpolymj.2016.12.027.

[2] Isola, M., Colucci, G., Diana, A., Sin, A., Tonani, A., Maurino, V., Thermal properties and decomposition products of modified cotton fibers by TGA, DSC, and Py–GC/MS, Polym. Degrad. Stab. 228 (2024) 110937. https://doi.org/10.1016/j.polymdegradstab.2024.110937.

[3] Montoya-Villegas, K. A., Licea-Claveríe, Á., Zapata-González, I., Gómez, E., Ramírez-Jiménez, A., The effect in the RAFT polymerization of two oligo(ethylene glycol) methacrylates when the CTA 4-cyano-4-(propylthiocarbonothioylthio) pentanoic acid is auto-hydrolyzed to its corresponding amide, J. Polym. Res. 26(3) (2019) 71. https://doi.org/10.1007/s10965-019-1718-4.
